# Global and Multiplexed Dendritic Computations under *In Vivo*-like Conditions

**DOI:** 10.1016/j.neuron.2018.08.032

**Published:** 2018-11-07

**Authors:** Balázs B. Ujfalussy, Judit K. Makara, Máté Lengyel, Tiago Branco

**Affiliations:** 1 MRC Laboratory of Molecular Biology, Cambridge, UK; 2 Laboratory of Neuronal Signaling, Institute of Experimental Medicine, Budapest, Hungary; 3 Computational and Biological Learning Lab, Department of Engineering, University of Cambridge, Cambridge, UK; 4 Department of Cognitive Science, Central European University, Budapest, Hungary; 5 Sainsbury Wellcome Centre, University College London, London, UK; 6 MTA Wigner Research Center for Physics, Budapest, Hungary

**Keywords:** dendritic integration, linear, nonlinear, hierarchical, input-output transformation, in vivo-like conditions, multiplexed, synaptic input, model, model fitting

## Abstract

Dendrites integrate inputs nonlinearly, but it is unclear how these nonlinearities contribute to the overall input-output transformation of single neurons. We developed statistically principled methods using a hierarchical cascade of linear-nonlinear subunits (hLN) to model the dynamically evolving somatic response of neurons receiving complex, *in vivo*-like spatiotemporal synaptic input patterns. We used the hLN to predict the somatic membrane potential of an *in vivo*-validated detailed biophysical model of a L2/3 pyramidal cell. Linear input integration with a single global dendritic nonlinearity achieved above 90% prediction accuracy. A novel hLN motif, input multiplexing into parallel processing channels, could improve predictions as much as conventionally used additional layers of local nonlinearities. We obtained similar results in two other cell types. This approach provides a data-driven characterization of a key component of cortical circuit computations: the input-output transformation of neurons during *in vivo*-like conditions.

## Highlights

• Understanding integration of complex synaptic inputs requires a model-based approach
• Hierarchical LN models accurately predict the responses of multiple cell types
• Linear subunits with a global dendritic nonlinearity achieve 90% prediction accuracy
• Analyses reveal a novel motif: multiplexing inputs into parallel processing channels

## Graphical Abstract

**Figure:**
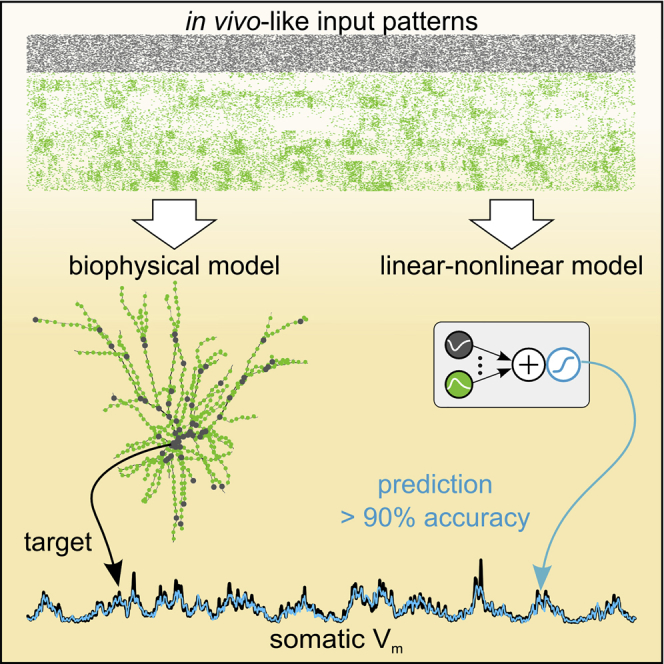

## Teaser

The input-output transformation of neurons under *in vivo* conditions is unknown. Ujfalussy et al. use a model-based approach to show that linear integration with a single global dendritic nonlinearity can accurately predict the response of neurons to naturalistic synaptic input patterns.

## Introduction

Cortical neurons receive and integrate thousands of synaptic inputs within their dendritic tree to produce action potential output. A large repertoire of biophysical mechanisms supporting a remarkable diversity of input integration properties has been described in dendrites, including synaptic saturation (Abrahamsson et al., 2012), dendritic spikes (Häusser et al., 2000), NMDA receptor (NMDAR) nonlinearities (Major et al., 2013), and interactions between excitation and inhibition (Palmer et al., 2012). A fundamental function of these mechanisms is to control the way single neurons convert spatiotemporal patterns of synaptic inputs to somatic membrane potential responses and, ultimately, action potential output. This input-output transformation at the level of individual neurons has a critical role in determining the population dynamics and computations performed by neural circuits (Rubin et al., 2015, Zador, 2000) and has been extensively investigated (Gerstner and Naud, 2009, Silver, 2010). Yet the mapping of dendritic inputs into somatic output under *in vivo* conditions remains poorly understood.

Our understanding of neuronal input integration remains limited because it is either based on data from *in vitro* experiments, studying neurons under highly simplified input conditions, or on *in vivo* approaches in which synaptic inputs were not observed or controlled, and thus a systematic characterization of the input-output transformation of neurons was not possible. *In vitro* approaches have been essential for characterizing the fundamental properties of dendritic integration by parametrically varying a small set of input features, such as the number, location, and timing of inputs in periodic trains of synaptic stimuli (Losonczy and Magee, 2006, Branco et al., 2010, Branco and Häusser, 2011, Makara and Magee, 2013). However, the input-output function of single neurons *in vivo* can in principle exhibit different properties than *in vitro* because of the high density and complexity of the synaptic input patterns characteristic of *in vivo* states and the high conductance regime they generate (London and Segev, 2001, Destexhe et al., 2003). In addition, recent experimental work has demonstrated that active dendritic conductances can substantially contribute to neuronal output *in vivo* (Xu et al., 2012, Lavzin et al., 2012, Palmer et al., 2014, Bittner et al., 2015, Takahashi et al., 2016), but it remains unclear how these active conductances change the neuronal input-output transformation. In principle they could produce a qualitative change (e.g., from linear to supralinear; Poirazi et al., 2003b, Polsky et al., 2004, Branco and Häusser, 2011, Makara and Magee, 2013), or they could simply change quantitatively the relative contributions of different synapses (Cash and Yuste, 1998, Magee, 2000, Häusser, 2001), leaving the neuron’s global computation unaffected. Thus, understanding the role of dendritic integration mechanisms in single-neuron computations requires both technical advances that allow experimental measurements of the spatiotemporal dynamics of synaptic activation across entire dendritic trees *in vivo* (Jia et al., 2010, Scholl et al., 2017) and new analysis methods for describing and quantifying dendritic and single-neuron computations.

To develop a new framework for analyzing single-neuron input-output transformations, we took inspiration from the domain of sensory processing, where statistical models have been successfully applied to predict neuronal responses to sensory stimuli with complex spatiotemporal structure *in vivo* (Ramirez et al., 2014). In these studies, the transformation of external inputs (e.g., visual images) to the neuronal response (e.g., of a visual cortical neuron) is expressed as a linear filtering step followed by a nonlinear transformation (linear-nonlinear or LN models, Pillow et al., 2008). This framework has the advantage that it allows the application of principled statistical methods to fit models directly to *in vivo* recordings and yields easily interpretable functional descriptions, two important features that are typically missing from approaches that involve fitting complex multicompartmental models to experimental data (Druckmann et al., 2007, Keren et al., 2009). However, in its standard form, the LN framework uses sensory stimuli as the main input to the model. As sensory input typically arrives several synapses upstream of the investigated cell, the recovered nonlinearity reflects a combination of the nonlinear processing steps at both the network and single-neuron levels (Antolík et al., 2016). Therefore, to isolate single-neuron input-output transformations, the LN framework needs a unique combination of features: inputs to the model must be the synaptic input received directly by the cell (Truccolo et al., 2010), the output must be the cell’s somatic response (Mensi et al., 2012, Ramirez et al., 2014), and a cascade of nonlinear input-output transformations must be allowed (Vintch et al., 2015, Freeman et al., 2015) to account for various forms of nonlinear processing in the dendrites and the soma.

Here, we have combined these features and show that hierarchical LN models (hLN) can accurately predict the subthreshold somatic response of neurons to complex spatiotemporal patterns of synaptic inputs. We use hLN models to study dendritic integration in biophysically detailed compartmental models of three neuron types that reproduce the main features of dendritic and somatic voltage activity recorded *in vivo* (Smith et al., 2013, Duguid et al., 2012, Grienberger et al., 2017). Surprisingly, we find that more than 90% of the somatic response can be accurately described by linear integration followed by a single global dendritic nonlinearity and that capturing *in vivo* membrane potential dynamics can require a conceptually new form of input processing, whereby dendritic subunits multiplex inputs into parallel processing channels with different time constants and nonlinearities. Our approach provides a quantitatively validated and intuitive description of dendritic information processing in neurons receiving large barrages of synaptic inputs and thus paves the way for obtaining accurate high-level models of input-output transformations in complex neurons—a critical step toward understanding the role of signal processing at the single-neuron level in the computations performed by neuronal circuits.

## Results

### Responses to Simple Stimuli Do Not Predict Responses to Complex Stimulation Patterns

**Figure 1:**
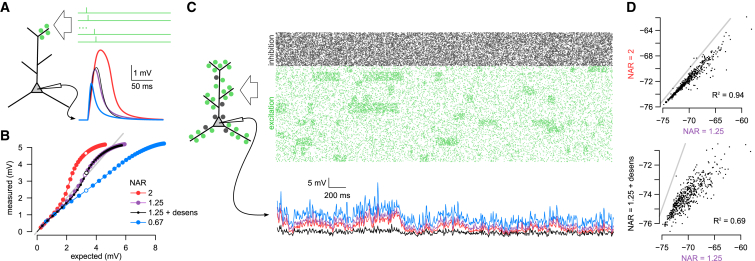
Responses to Simple Stimuli Do Not Predict Responses to Complex Stimulation Patterns (A) Illustration of a typical *in vitro* protocol stimulating a small number of neighboring synapses (left) using a fixed temporal delay between stimuli (top right) while recording the somatic membrane potential response (bottom right). Somatic responses of four model neurons are shown (colors as in B and C) to stimulus sequences with the number of stimuli chosen such that the expected responses of all neurons were near-identical (see open circles in B). (B) Measured response amplitudes in different model neurons (colors) to the stimulation of 1–40 neighboring synapses at 1 ms intervals as a function of the response amplitude expected from linear integration. Open circles indicate simulations shown in (A) (expected amplitude ∼3.3 mV); gray line shows identity line (exactly linear integration). Models differed in the NMDA-to-AMPA ratio (NAR) of their glutamatergic synapses, resulting in qualitatively different modes of dendritic integration: supralinear (red, NAR = 2), approximately linear (purple and black, NAR = 1.25) and sublinear (blue, NAR = 0.67). Only the model shown in black included desensitization of NMDA receptors (with a fast and a slow desensitized state of time constants 40 ms and 220 ms). (C) Responses of the same four model neurons as shown in (A) and (B) to sustained *in vivo*-like inputs with complex spatiotemporal structure. Top: input spike trains arriving at excitatory (green) and inhibitory synapses (gray). Bottom: the somatic membrane potential in the four neurons in response to the stimulus shown above (color code as in A and B). (D) Correlation between the responses of selected pairs of model neurons shown in (C): neuron with NAR = 2 (red) versus neuron with NAR = 1.25 (purple, top) and the neuron with NAR = 1.25 and desensitizing NMDA receptor (black) versus the neuron with NAR = 1.25 but without NMDA desensitization (purple, bottom). Gray lines show identity lines. Note the lack of relationship between correlations under *in vivo*-like conditions (D) and similarity of responses to *in vitro* stimulation protocols (A and B).

To illustrate the potential shortcomings of the most common approach for characterizing dendritic integration (Polsky et al., 2004, Losonczy and Magee, 2006, Branco et al., 2010, Abrahamsson et al., 2012, Makara and Magee, 2013), we used a previously validated multicompartmental biophysical model of a L2/3 cortical pyramidal cell (Smith et al., 2013) and recorded the somatic membrane potential response while stimulating the cell with inputs that were either similar to those typically used in *in vitro* experiments or resembled naturalistic patterns expected to emerge *in vivo*. First, we used regular trains of synaptic input (up to 40 glutamatergic synapses, 1 stimulus per synapse at 1 ms inter-stimulus intervals; Figure 1A). We characterized the input-output function of the neuron by comparing the magnitude of the measured response with that expected from linearly summing the responses to individual synaptic stimuli (Figure 1B). We then varied the NMDA-to-AMPA maximal conductance ratio (NAR) in the model. In line with previous results (Behabadi et al., 2012), higher NAR resulted in a marked increase in the supralinearity of the input-output transformation as measured by this analysis method (Figure 1B, compare red, purple, and blue). However, the same model neurons showed very little difference in their behavior when stimulated with complex patterns expected to emerge *in vivo* (600+ glutamatergic and 200+ GABAergic synapses, stimulated at rates dynamically fluctuating between 5 and 20 Hz and between 20 and 30 Hz, respectively; Figure 1C, see also STAR Methods). Apart from a slight tonic offset, the somatic response of the different models was very highly correlated (Figure 1D, top). Conversely, we also compared two model neurons that differed only in the kinetics of NMDAR desensitization (Figures 1A and 1B, purple versus black). While the *in vitro*-like stimulation protocol revealed no difference between the two models, *in vivo*-like stimulation produced markedly different responses (Figure 1C) that were only weakly correlated (Figure 1D, bottom). This was due to *in vivo*-like patterns frequently activating the same synapse more than once and leading to differences in the NMDAR conductance availability between the two neurons.

These two examples demonstrate that due to the dynamic and sustained nature of synaptic activation under *in vivo*-like input patterns, differences and similarities across different cells and conditions cannot be readily predicted from responses to simple stimulation protocols (London and Segev, 2001). Moreover, even under sustained stimulation, the degree to which dendritic processing appears nonlinear can depend on whether the stimulus is stationary, with constant firing rates (Poirazi et al., 2003b), or whether it shows fluctuations spanning the full dynamic range of the neuron (Figure S1). This motivated us to characterize neuronal input-output transformations during complex, fluctuating spatiotemporal input patterns directly. As the measured-versus-expected method does not generalize to this input regime, we developed a new model-based analysis technique to estimate the input-output function of single neurons.

### Fitting the Input of a Biophysical Model to *In Vivo* Dendritic Recordings

**Figure 2:**
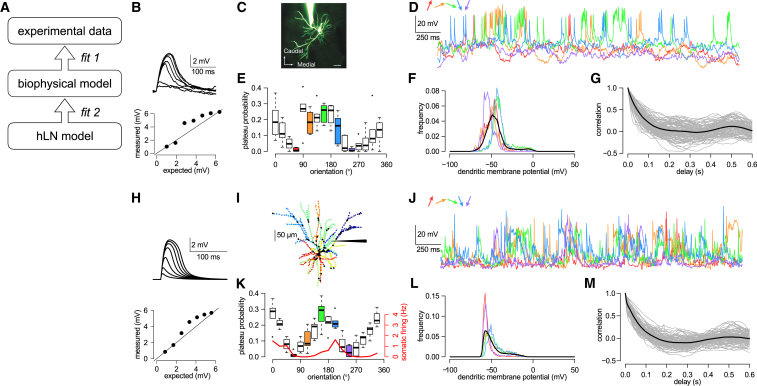
Fitting the Input of a Biophysical Model to *In Vivo* Dendritic Recordings (A) Logic of the approach. We first matched the dendritic and the somatic response of a detailed biophysical model to *in vivo* data (fit 1). This step was required because there are no experimental measurements of the spatiotemporal activation profile for all synapses of a neuron *in vivo* and its corresponding output. Next, we tuned the parameters of the phenomenological hLN model to match the somatic membrane potential time course of the biophysical model in response to known synaptic inputs (fit 2). (B) Experimental data showing nonlinear dendritic integration in a layer 2/3 pyramidal neuron *in vitro* (reanalyzed from Branco et al., 2010). Top: somatic responses to 1–7 glutamate uncaging events at 1 ms intervals on a single dendritic branch. Bottom: measured response amplitudes as a function of the response amplitude expected from linear integration. (C) Two-photon microscopy image (maximum intensity projection) of an Alexa Fluor 594-filled layer 2/3 pyramidal neuron in the mouse visual cortex during a dendritic patch-clamp recording *in vivo* (scale bar, 20 μm). Reproduced from Smith et al. (2013). (D) Examples of membrane potential recordings from a single dendrite in response to differently oriented drifting gratings (colors). Experimental data are from Smith et al. (2013). The same dendrite is analyzed in (E)–(G). (E) Orientation tuning of plateau potentials in the dendritic branch. Boxplots show median, quartiles, and range of data; open circles indicate outliers. (F) Histogram of the dendritic membrane potential for different sample input orientations (colors as in D and E) and the average across all different orientations (black). (G) Auto-correlation of the dendritic membrane potential (gray, individual traces for each orientation and repetition; black, average). (H) Nonlinear dendritic integration in a biophysical model layer 2/3 pyramidal neuron (analyzed as in B). (I) Morphology of a reconstructed L2/3 pyramidal neuron and the distribution of inhibitory (black dots) and excitatory synapses (synapses with the same color received correlated inputs). Schematic electrode points to dendrite analyzed in (J)–(M). (J) Membrane potential traces recorded in a model dendritic branch in response to sustained, *in vivo*-like inputs corresponding to different orientations (colors as in D). (K–M) Orientation tuning (K), membrane potential histogram (L), and auto-correlation (M) of the model dendrite. Colors and symbols are as in (E)–(G); red line in (K) shows somatic orientation tuning. Boxplots in (K) show median, quartiles, and range of data; open circles indicate outliers.

Studying the contribution of dendritic integration to the input-output mapping performed by single neurons *in vivo* requires observing both the inputs and the output simultaneously, with high spatiotemporal precision. Although the combination of high-resolution two-photon imaging techniques with *in vivo* patch-clamp recordings will likely be able to meet this demand in the near future (Grienberger and Konnerth, 2012), such data are not yet available. Therefore, we took a two-step modeling approach (Figure 2A): first, we implemented a detailed biophysical model neuron that reproduced dendritic activity recorded experimentally *in vivo* (Figure 2A, fit 1); second, we delivered a wide range of spatiotemporal input patterns to the biophysical model and used the known inputs and outputs of this model to estimate the single neuron input-output function using hLN models (Figure 2A, fit 2). Importantly, the biophysical model was matched as closely as possible to *in vivo* data to ensure that the nonlinearities measured *in vivo* were also expressed by our model.

We first implemented this approach in layer 2/3 neocortical pyramidal neurons in the visual cortex (Figure 2C; Smith et al., 2013) because their biophysical properties (Larkum et al., 2007, Branco et al., 2010, Branco and Häusser, 2011) and *in vivo* somatic (Poulet and Petersen, 2008, Smith et al., 2013, Petersen and Crochet, 2013, Polack et al., 2013) and dendritic (Smith et al., 2013, Palmer et al., 2014) activity have been well characterized. In particular, *in vitro* stimulation paradigms have revealed strongly supralinear dendritic integration in these cells (Figure 2B; Branco et al., 2010), and *in vivo* experiments have shown dendritic plateau depolarizations in response to visual stimulation (Figure 2D and 2E; Smith et al., 2013) that result from the recruitment of dendritic nonlinearities (Palmer et al., 2014) and enhance the selectivity of orientation tuning (Smith et al., 2013).

To replicate input integration in layer 2/3 neurons under *in vivo*-like conditions, we used a previously validated biophysical model that reproduced dendritic nonlinearities observed *in vitro* (Figure 2H; Branco et al., 2010, Smith et al., 2013) and tuned the input statistics to reproduce the dendritic and somatic membrane potential dynamics measured experimentally *in vivo*. Specifically, we included 600+ excitatory (with AMPA and NMDA receptors) and 200+ inhibitory synapses (with GABA-A receptors), where the majority of the inhibitory synapses were located near the soma and all other synapses were distributed uniformly throughout the entire dendritic tree (Figure 2I). Excitatory synapses were organized into a number of ensembles with distinct orientation and phase preferences. Inputs belonging to the same ensemble underwent coordinated stimulus-dependent switches between a background and an elevated rate as well as slow, stimulus-independent fluctuations, and they co-clustered on contiguous stretches of the dendritic tree. This clustering of co-active presynaptic inputs on the same dendritic branch facilitated the induction of dendritic plateaus (Takahashi et al., 2012, Scholl et al., 2017), while inhibitory synapses formed a single ensemble with a rate that tracked the overall activity of excitatory inputs. Input parameters were varied systematically to optimize the match to three experimentally measured summary statistics of dendritic membrane potential recordings: the overall plateau probability, the decay time constant of autocorrelations, and the distribution of membrane potential values. In addition, throughout our analyses we tested and confirmed that our main results were robust to variations in input statistics.

The best-fitting biophysical model had 13 excitatory ensembles (Figure 2I) and a relatively low rate of background excitation (5 Hz) and inhibition (20 Hz), alternating with elevated excitatory (20 Hz) and inhibitory synaptic activity (30 Hz; Haider et al., 2013), which could reproduce the experimentally observed bimodal nature of dendritic membrane potential distributions (Figures 2F and 2L). Although excitation and inhibition were balanced overall in our model, during periods of elevated synaptic activity the differential spatial distribution of excitatory and inhibitory synapses caused sufficient dendritic depolarization to elicit NMDAR-dependent plateau potentials (Figure 2J). Due to the clustering of stimulus-tuned inputs in our model, the probability of these plateaus showed clear orientation tuning matching the experimental data (Figures 2E and 2K), and their duration was such that autocorrelations also decayed on the experimentally observed timescales (Figures 2G and 2M). The soma had a peak firing rate between 1 and 2 Hz and also showed orientation tuning (Figure 2K, red) similar to experimental data (Polack et al., 2013, Smith et al., 2013). Having extensively validated this biophysical model on experimental data, we next used it as a test-bed to analyze dendritic processing under *in vivo*-like conditions.

### A Hierarchical Linear-Nonlinear Model of Dendritic Integration

**Figure 3:**
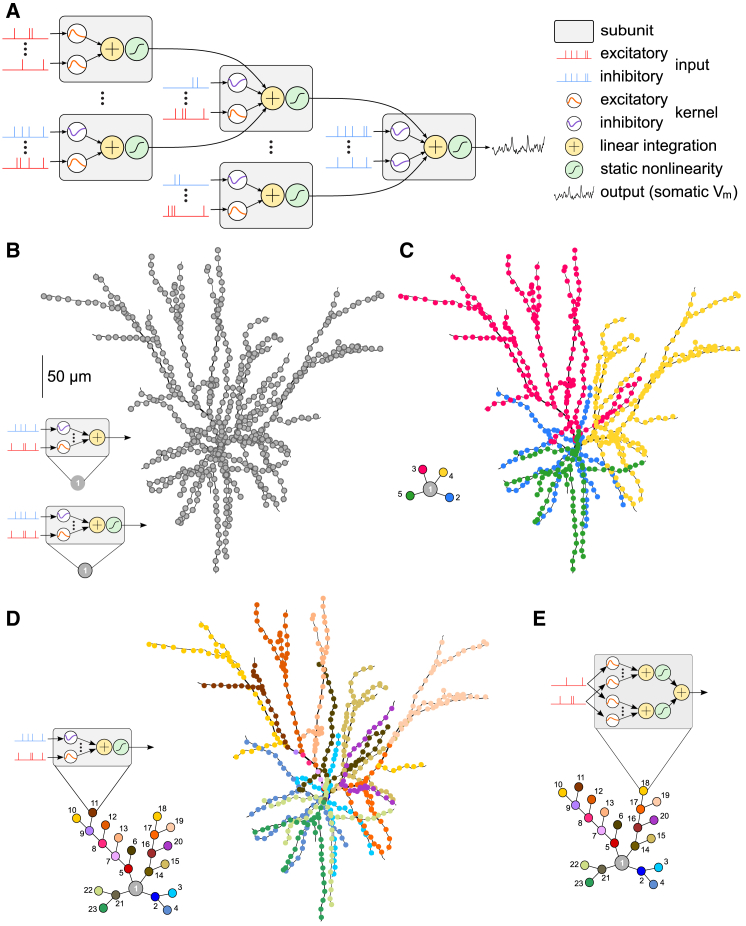
A Hierarchical Linear-Nonlinear Model of Dendritic Integration (A) Schematic of an hLN model with five subunits. Each subunit (gray boxes) receives input from a number of excitatory (red) and inhibitory spike trains (blue), filtered by positive (orange) or negative synaptic kernels (purple). The filtered inputs are summed linearly (yellow) and passed through a sigmoidal nonlinearity (green) before being integrated at the next stage of the hierarchy. (B–E) hLN model architectures of increasing complexity (left) capturing synaptic integration in the biophysical model (right). Each colored circle of an hLN model corresponds to an individual subunit with input spike trains from a subset of synapses (correspondingly colored dots for excitatory synapses shown on the biophysical model morphology) and an output nonlinearity (except for the single subunit of the model in B, top, see also insets). Gray circles correspond to output subunits. Models from (B) to (D) expand the depth of the hierarchy from a 1-subunit (1-layer) “point neuron” model (B) to a 23-subunit (6-layer) model (D). The model in (E) expands the breadth of the hierarchy by multiplexing synaptic inputs such that each input spike train feeds into two parallel input channels with different synaptic kernels and nonlinearities (inset shows the schematic of a single, multiplexing subunit).

To capture the high-dimensional and potentially complex input-output mapping of single neurons under *in vivo*-like conditions, including the effects of nonlinear dendritic processing, we adapted a hierarchical extension of the widely used LN model (Vintch et al., 2015, Freeman et al., 2015). In our hierarchical LN (hLN) model, the input-output transformation of the cell was formalized as a hierarchy of simple subunits (Figure 3A, gray boxes), such that inputs to the same subunit (Figure 3, red and blue spike trains) were first linearly integrated temporally (using a mixture of standard alpha function synaptic kernels; Figure 3A, orange and purple) as well as spatially (Figure 3A, yellow), and a separate sigmoidal nonlinearity acted on the output of each subunit (Figure 3A, green) before it was linearly combined again with the outputs of other subunits. By shifting the threshold of the nonlinearity relative to the input distribution, the nonlinear transformation could be made either effectively sub- or supralinear and therefore capture biophysical processes such as driving force saturation or NMDAR activation.

The form of this model was motivated by previous studies, suggesting a similar compartmentalization of dendritic nonlinearities into such functional subunits that include the effects of synaptic processing (the kernels) and nonlinear input integration (the combination of summation and an instantaneous nonlinearity) (Poirazi et al., 2003a, Polsky et al., 2004). However, while previous work has considered a one-to-one correspondence between functional subunits and individual dendritic branches, the hLN framework is more flexible in how its subunit architecture maps onto the morphology of the cell. We thus created subunits that corresponded to contiguous sections of the dendritic tree, including a smaller or larger number of connected branches, depending on the complexity of the hLN model (i.e., more complex models included a higher number of smaller subunits; Figures 3B–3D, see also below).

To dissect the contributions of dendritic integration to the somatic response when fitting the hLN model, we isolated the subthreshold somatic membrane potential in the biophysical model by removing its action potential generation mechanism (Figure 3A, black trace to the right). Thus, despite its apparent soma-like position in the subunit architecture, the final and therefore global nonlinear subunit in the hLN model corresponded to dendritic nonlinear mechanisms activated over the entire dendritic tree, which could be sub- or supralinear depending on the dominant biophysical mechanism. (See Figure S4 for results with fitting both the sub- and suprathreshold behavior of the somatic membrane potential.)

The parameters of the hLN model (amplitude and time constants of excitatory and inhibitory synaptic kernels, thresholds of the nonlinearities, and the output weight of each subunit) were fit to simultaneously recorded input-output data (synaptic input pattern and respective somatic membrane potential obtained with the biophysical model) using principled, maximum-likelihood-based statistical techniques (STAR Methods). We rigorously validated both our statistical methods for model fitting and the ability of the hLN model class to correctly capture the integration of spatially distributed inputs, despite its drastic discretization of the cell’s morphology into a small number of independent subunits (Figures S2–S3).

### Global Input-Output Transformation in L2/3 Pyramidal Neurons

**Figure 4:**
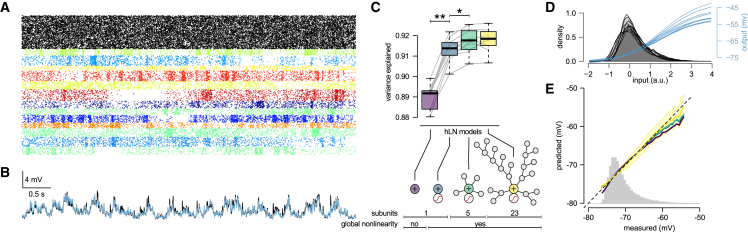
Global Input-Output Transformation in L2/3 Pyramidal Neurons (A) Presynaptic inhibitory (black) and excitatory input spike trains (colors, as in Figure 2I) used for fitting the biophysical model to experimental data (Figure 2). (B) The somatic membrane potential in the biophysical model (black) and the output of the hLN model with linear integration and a global nonlinearity (blue) in response to the input shown in (A). Parameters of the biophysical model and the inputs were identical to that shown in Figure 2, except that somatic active conductances were removed. (C) Prediction accuracy (variance explained) of hLN models with increasing complexity. Bottom shows the architectures of different hLN models and table summarizing their main properties (*cf.*Figures 3B–3D). Gray lines show individual datapoints and boxplots show median, quartiles, and range of the data. ^∗^p < 0.005, ^∗∗^p < 10^−7^. (D) The nonlinearity of the input-output transformation (blue) and the distribution of linearly integrated synaptic inputs (gray) in the one-subunit model for ten different simulations. (E) Mean of model predictions as a function of the measured response (colored lines). Gray histogram shows the distribution of the measured response; black dashed diagonal shows identity line. Yellow shaded area indicates the standard deviation of the 23-subunit model’s prediction.

We formalized alternative hypotheses about the functional form of dendritic input integration by generating hLN models with increasingly complex architectures that differed in the number of nonlinear dendritic subunits (Figures 3B–3D) and in whether the final subunit performed linear or nonlinear integration (Figure 3B). The architectures of these hLN models followed the morphology of the biophysical model and its input distribution as much as possible given the number of available LN subunits (*cf.* Figure 2I). We then fitted each of these models to the same dataset generated by the biophysical model (Figure 2), such that the inputs were the synaptic input patterns received by the biophysical model (Figure 4A) and the output was its somatic membrane potential (Figure 4B, black). We quantified hLN model accuracy by the fraction of variance of the somatic membrane potential it explained in cross-validation, on a held-out test dataset (Figure 4C).

As expected, because we used a sequence of nested models (i.e., the more complex models always included the simpler ones as special cases), successful fitting of these models led to monotonically increasing accuracy in predicting the biophysical model’s behavior (Figure 4C). Nevertheless, we found that models with a single subunit, and thus performing linear processing, with (Figure 3B, bottom) or without a global nonlinearity (Figure 3B, top), already explained 90% variance (Figures 4B and 4C, purple and blue). When included, the best-fit global nonlinearity was supralinear (Figure 4D), suggesting that at high input rates the effects of NMDA activation dominate over those of driving force saturation in the dendrites. Introducing more subunits and hence more local nonlinear processing in the hLN model (Figures 3C and 3D) increased its accuracy only slightly, but significantly (p < 0.005), to 91.5% (Figure 4C, green and yellow). In particular, adding more dendritic subunits increased the ability to predict large somatic depolarizations (Figure 4E) but did not reduce the overall variance of hLN predictions.

To test the generality of these findings, we re-fitted the same hLN models to data obtained by stimulating the biophysical model with a wider range of spatiotemporal input patterns (Figures S5–S7). We systematically varied three parameters known to influence the recruitment of dendritic nonlinearities: input synchrony, input firing rates, and the number of synapses organized into functional clusters (synapses with correlated inputs). We found that for all cases tested, linear models accounted for at least 80% of variance and for at least 90% within the physiologically feasible regime (based on the three summary statistics of dendritic membrane potential recordings described above). Multiple layers of independent nonlinear subunits improved predictions by only 2% at most. Critically, given the 80%–90% performance of hLN models with only a single global nonlinearity, the inclusion of more complex architectures or local nonlinear processes could not have achieved more than 10%–20% improvement. Thus, these results suggest that the input-output transformation of L2/3 pyramidal cell dendrites can be well described by linear processes followed by a global nonlinearity.

### Global Input-Output Transformation in Other Cell Types

To further corroborate our findings, we repeated the same analyses using biophysical models of two other cell types whose natural input patterns have been reasonably well characterized: a cerebellar granule cell and a CA1 pyramidal neuron.

First, we simulated a detailed biophysical model of a cerebellar granule cell and fitted hLN models to its subthreshold somatic membrane potential (Figure S8). The biophysical model reproduced the statistics of both the known excitatory and inhibitory inputs as well as the output firing rate dynamics of the cell during various *in vivo* experimental conditions. Granule cells are electrotonically compact: their dendrites do not effectively compartmentalize their nonlinearities and are thus unlikely to implement multilayered functional architectures. In agreement, the best hLN model for the granule cell achieved 95% accuracy with a single layer and global linear integration.

Second, we also fitted hLN models to a multicompartmental biophysical model of a CA1 pyramidal neuron endowed with dendritic NMDA- and Na^+^-spike-generating mechanisms (Figure S9). To generate *in vivo*-like input, we modeled 2,000 place cells modulated by the theta oscillation and exhibiting phase precession (simulating an animal moving along a linear track; O’Keefe and Recce 1993), as well as 200 interneurons also modulated by the theta oscillation. As for the previous two cell types, we found that > 90% variance of the somatic response of the CA1 cell was captured by a hLN model including a single subunit with a global nonlinearity, though (as for L2/3 cells) a 2-layer (5-subunit) hLN model significantly outperformed the 1-layer model, achieving above 95% accuracy.

Taken together, these results show that in multiple cell types, synaptic input processing can be described to high accuracy by a linear process with multiple kernels. In a simple cell such as cerebellar granule cells, the linear component alone achieves 95% accuracy, but for more complex cells, the highest prediction accuracy requires a global dendritic nonlinearity or even a second processing layer.

### Active Dendritic Conductances Change the Properties of Linear Input-Output Transformations

**Figure 5:**
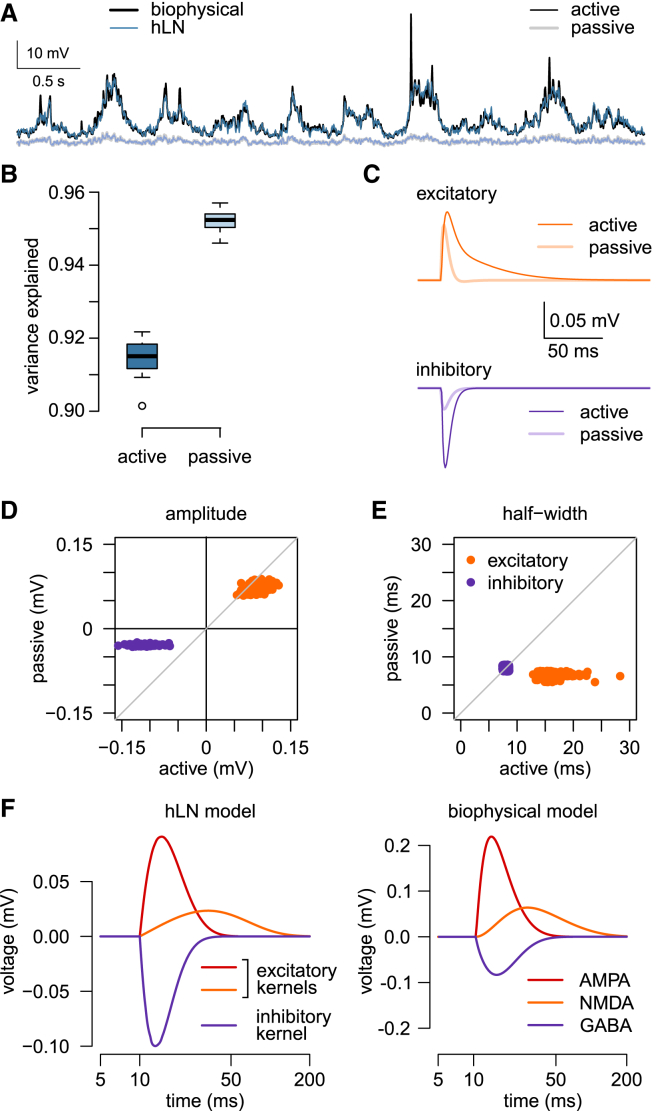
Active Dendritic Conductances Change the Properties of Linear Input-Output Transformations (A) Somatic membrane potential in the active (black) and passive (gray) biophysical neuron model to *in vivo*-like stimulation (as in Figures 2 and 4) together with the prediction of the hLN model with a single LN subunit (dark and light blue, respectively). (B) Variance explained by the hLN model for the active (dark blue) and passive cell (light blue). Boxplots show median, quartiles, and range of ten independent simulations; open circle indicates an outlier. (C) Average excitatory (top, orange) and inhibitory (bottom, purple) synaptic kernels for fitting the responses of the active (dark colors) or passive cell (light colors). (D and E) Amplitude (D) and half-width (E) of individual excitatory (orange dots) and inhibitory (purple dots) synaptic kernels for fitting the active versus the passive model. Gray diagonals show identity. (F) Average elementary synaptic kernels recovered by the hLN model when fitting *in vivo*-like input-output mapping (left) and average synaptic responses in the (active) biophysical model in response to individual stimuli (right). Note different scales on y axes: quantitatively, the amplitude of the estimated excitatory (inhibitory) kernels of the hLN model fitting *in vivo*-like data were smaller (larger, respectively) than the PSPs in the biophysical model due to the effects of the high conductance state on membrane properties. Note logarithmic time axes.

Although our analyses so far have shown that linear processing can account for ∼90% of the somatic membrane potential variance in neurons with nonlinear dendrites, this does not imply that active dendritic mechanisms have a negligible contribution to input integration. Instead, active dendritic nonlinearities may contribute by changing the linear component of the cell’s input-output transformation (Cash and Yuste, 1999). To investigate this possibility, we compared hLN models with a single LN subunit (Figure 4C, blue) that were fitted either to our standard L2/3 biophysical model (Figure 5A, black) or to a passive variant that was identical in all parameters except that it did not have any active conductances, including NMDARs (Figure 5A, gray). We found that, as expected, a 1-subunit model provided a slightly better fit to the passive than to the active cell (Figures 5A and 5B, light and dark blue, 95% versus 91.5%, p < 0.001). However, the synaptic kernels underlying these fits were drastically different: excitatory kernels were larger and longer-lasting in active dendrites (Figure 5C, top; Figures 5D and 5E, orange), while inhibitory kernels became larger but remained similar in their time courses (Figure 5C, bottom; Figures 5D and 5E, purple). The differences in excitatory kernels mainly reflected the recruitment of NMDAR currents in active dendrites, which also changed the inhibitory kernels due to an increase in driving force caused by larger overall dendritic depolarization. Thus, in addition to adding a nonlinear component to the input-output transformation when synaptic input is highly frequent and spatiotemporally correlated, a critical effect of active dendritic conductances is to change the linear integration properties of the neuron.

As the accuracy of linear hLN models was highest when synaptic kernels were linear mixtures of elementary alpha functions (see STAR Methods), we wondered whether the particular kernels found during fitting these models to data provided additional biological insight. We found that the best-fit elementary kernels came to closely resemble the individual postsynaptic potentials (PSPs) corresponding to the three different receptor channels in the biophysical model (AMPA, NMDA, and GABA) (Figure 5F). We obtained similar results with the cerebellar granule cell model, where the kernels of the hLN model recovered all four different PSPs of the biophysical model (AMPA, NMDA, fast and slow GABA; Figure S8). The ability to recover “ground truth” in these cases highlights a strength of the hLN approach: it allows the joint estimation of all parameters of the functional architecture of input-output transformations in a cell (kernels, nonlinearities, and their hierarchy) during *in vivo*-like conditions, without the need to conduct piecemeal minimal stimulation experiments and simulations, and analyses that may ultimately not generalize well to the *in vivo* case.

### Input Multiplexing

An advantage of formalizing the input-output mapping of single neurons with hLN models is the possibility of testing functional architectures that have not been considered previously but that follow logically from this model class and may present viable descriptions of the effects of nonlinear dendrites. In particular, while in previous studies the transition from single- to multiple-subunit hierarchies has exclusively been considered to imply an increase in the depth of the architecture (Häusser and Mel, 2003), we also considered whether increasing its breadth may also increase its predictive power. To test this, we multiplexed every synaptic input to each subunit into two channels with independent excitatory and inhibitory kernels and nonlinearites, thus allowing input integration by two potentially distinct nonlinear processes in parallel (Figure 3E). Specifically, we wondered whether the advantage gained from including synaptic kernels with multiple timescales (to capture combinations of fast and slow synaptic processes such as AMPA and NMDAR conductances) could be further extended by also allowing different nonlinearities to act on these different timescales.

**Figure 6:**
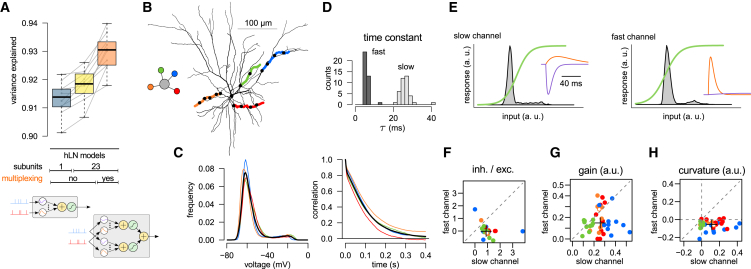
Input Multiplexing (A) Prediction accuracy (variance explained) of hLN models with increasing complexity, including multiplexing (orange). Blue, yellow: same as in Figure 4C, shown here for reference. Gray lines show individual data points, and boxplots show median, quartiles, and range of the data. Table in middle summarizes the main properties of different hLN models (*cf.*Figure 4C); bottom illustrates difference between non-multiplexing (left) and multiplexing subunits (right, *cf.*Figures 3D and 3E). (B) Biophysical cell model with four dendrites stimulated (colored) and the architecture of the hLN model fitted to its responses (inset). (C) Membrane potential distributions (left) and autocorrelograms (right) in individual dendrites (colors as in B) and their average (black). (D) Distribution of excitatory time constants in the two input channels show clear bimodality (dark versus light gray) across the four subunits and ten independent fits. (E) Properties of the two input channels (left, slow; right, fast) in a representative subunit. Gray histograms indicate distributions of excitatory synaptic inputs after temporal filtering with the corresponding synaptic kernels; green lines indicate output nonlinearities of the input channels. Insets show independently fitted excitatory (orange) and inhibitory (purple) synaptic kernels. As the inhibitory inputs are well captured by a single kernel targeting the slow channel, the inhibitory kernel in the fast channel is prone to overfitting and can take small, positive amplitudes. (F–H) Ratio of inhibitory to excitatory synaptic kernel amplitudes (F) and the slope (G) and curvature (H) of the output nonlinearity (averaged under the filtered input distribution; see gray histograms in D) in fast versus slow input channels across subunits (colors as in B) and ten independent fits. Negative or positive curvature in (H) implies sublinear or supralinear integration, respectively. Crosses in (F)–(H) indicate population medians; empty circles correspond to the examples shown in (E).

We found that input multiplexing could substantially improve model accuracy (Figure 6A). In particular, the addition of multiplexing to hLN architectures (Figure 6A, orange versus yellow) increased predictive power more than expanding the number of subunits from 1 to 23 (Figure 6A, yellow versus blue). To understand what biophysical aspects of neuronal input integration were captured by multiplexing, we analyzed a simplified case, in which only four dendrites in a L2/3 neuron were stimulated (Figure 6B) with patterns similar to those producing realistic dendritic membrane potential distributions and autocorrelograms (Figure 6C; *cf.* Figure 2). We then compared the best-fit parameters of the two input channels for each subunit (Figures 6D–6H) and found that the two channels were markedly different in three aspects: (1) their “speed,” i.e., the time constant with which they integrated excitatory inputs (5.9 ± 1.4 ms versus 26.2 ± 3.2 ms; Figures 6D and 6E); (2) the balance of excitatory versus inhibitory inputs, with inhibitory synapses having a much larger weight in the slower channel (Figures 6E and 6F); and (3) the location of the input distribution relative to the threshold of the sigmoidal nonlinearity (Figure 6E). This resulted in the slower channel applying higher average gain (Figure 6G) and a more strongly supralinear transformation to its inputs than the fast channel (Figure 6H). These results are compatible with the fast channel capturing AMPA receptor-like properties, while the slow channel captures NMDA and GABA receptor activation during periods of high input frequency, and thus demonstrate the power of multiplexing for incorporating complex biophysical properties into a high-level descriptive model.

## Discussion

We have introduced a novel model-based approach for analyzing dendritic integration and describing the input-output transformation of single neurons with complex dendritic processing receiving *in vivo*-like input patterns. A major advance of this work is the development of the new analysis methods based on a flexible and powerful model class, the hLN model, that provides a compact mathematical characterization of the input-output transformations of individual neurons and can be efficiently fitted to data. We used our approach to analyze integration of direction-selective inputs in a biophysical model of a L2/3 pyramidal neuron in visual cortex and found that dendrites of L2/3 pyramidal cells receiving large barrages of synaptic input do not act as independent computational subunits, but instead the entire dendritic tree behaves as a single LN unit (as in Figure 3B). This contrasts with the conclusion from experimental and modeling findings in simple-input regimes, where single dendritic branches have been suggested to perform independent nonlinear computations (as in Figures 3C and 3D; Poirazi et al., 2003b, Polsky et al., 2004, Losonczy and Magee, 2006). Our results were replicated under a wide range of input patterns and in two further cell types: cerebellar granule cells and hippocampal CA1 pyramidal cells. Moreover, our analysis also showed that multiplexing inputs to parallel (slow and fast) processing channels within computational subunits is a form of nonlinear dendritic processing that can be equally important as the classically considered serial hierarchy of subunits (Häusser and Mel, 2003). Further biological insights provided by our work are that, under *in vivo*-like conditions, the dominant effect of high input rates in L2/3 neurons is to recruit NMDARs and generate supralinear integration, instead of the commonly assumed global conductance increase and driving-force saturation, which would lead to sublinearity (London and Segev, 2001, Destexhe et al., 2003), and that the main contribution of voltage-dependent mechanisms such as NMDARs, in neurons under these conditions, is to predominantly change the gain of linear integration instead of introducing a strong nonlinearity.

### Synaptic Integration under *In Vivo*-like Input Conditions

Previous theoretical work on neuronal input processing during *in vivo*-like conditions mainly focused on the increase in input conductance caused by persistent synaptic bombardment (the “high conductance state”) and analyzed its effects on the efficacy of synaptic inputs (Destexhe et al., 2003) and on events such as the initiation and propagation of dendritic spikes (Rudolph and Destexhe, 2003, Williams, 2004, Jarsky et al., 2005, Farinella et al., 2014). While these studies highlighted important differences in synaptic integration between the quiescent and the *in vivo*-like states and provided a means to evaluate the processing of complex input patterns, they did not describe the input-output transformation of individual neurons during *in vivo*-like input conditions. The approach we have developed provides a principled way of achieving this and can be applied to data from both compartmental models and from experiments simultaneously recording synaptic input patterns over the entire dendritic tree and somatic membrane potential, once these become available (Grienberger and Konnerth, 2012).

### Mathematical Models of Dendritic Processing

Developing compact mathematical characterizations of the input-output transformations of individual neurons is a long-standing challenge (Gerstner and Naud, 2009, Poirazi et al., 2003b) that is a critical step toward understanding the population dynamics and computations that emerge at the level of neural circuits (Ahmadian et al., 2013, Rubin et al., 2015, Hennequin et al., 2018). However, classical principled methods for distilling simplified single-neuron models are only formally valid for electrotonically compact neurons, in which the contribution of dendritic processes for synaptic integration is minimal, and for neurons with passive dendrites that lack voltage-dependent conductances. Similarly, due to the vast complexity of dendritic nonlinearities and the current lack of a formalization of their contributions to single-neuron computations, the majority of theories of network-level computations either rely on single-compartmental models (Dayan and Abbott, 2001) and thereby ignore the role of dendrites, assume linear dendritic processing (Cook and Johnston, 1997), or make very specific assumptions about the form of dendritic nonlinearities based on largely qualitative arguments (e.g., coincidence detection; Pecevski et al., 2011, Kaifosh and Losonczy, 2016).

The hLN framework developed here offers a principled way of estimating the contribution of nonlinear dendritic processing to the response of neurons and incorporating it efficiently in single-neuron models designed for network simulations. This approach has its roots in system identification (Wu et al., 2006, Lazar and Slutskiy, 2015) applied to modeling of visual signal processing in the retina (Schwartz et al., 2006) or of neuronal responses to somatic and dendritic current injections (Cook et al., 2007). While the focus of system identification in systems neuroscience has mainly been on the mapping from an analog stimulus (e.g., visual pixel intensities or input currents) to the binary spiking response of the recorded neuron (Schwartz et al., 2006), we derived a mapping from binary presynaptic spike trains to the analog somatic membrane potential.

The hLN model framework and level of analysis is complementary to biophysical modeling of single neurons: hLN models provide a compact and intuitive description of the input-output transformation implemented by the neuron, but they lack detailed mechanistic insight. In contrast, biophysical models can reveal the physiological processes underlying signal integration and propagation in neurons, but the overall picture of how the neuron transforms information often remains obscure (Herz et al., 2006). Moreover, biophysical models accurately matched to data have the potential to generalize across different input conditions (Druckmann et al., 2011), but parameter tuning in these models is challenging (Huys et al., 2006, Friedrich et al., 2014) and simulations are computationally very expensive. Conversely, while the accuracy of hLN models is limited to the specific input regime within which they were fitted, they are very efficient to simulate and fit data, naturally lending themselves to be integrated into large-scale network simulations.

### Linear Integration

Several *in vitro* (Golding and Spruston, 1998, Schiller et al., 1997, Schiller et al., 2000, Larkum et al., 1999, Branco et al., 2010, Makara and Magee, 2013, Vervaeke et al., 2012, Abrahamsson et al., 2012, Hoffman et al., 1997, Urban and Barrionuevo, 1998, Hu et al., 2010) and *in vivo* (Smith et al., 2013, Lavzin et al., 2012, Xu et al., 2012) experimental studies have established that dendritic integration is a fundamental component of input processing in neural circuits. The hLN approach described here is a tool for analyzing how dendritic mechanisms change the input-output transformations of single neurons. By evaluating the computations performed by a biophysical model of a cortical neuron receiving *in vivo*-like inputs, we have found that only 10% of the response variance can be attributed to local nonlinear dendritic processes when the input statistics produced membrane potential profiles that matched *in vivo* recordings (Smith et al., 2013). This is consistent with previous estimates of the contribution of nonlinear processing to the somatic membrane potential responses of cortical cells (Jolivet et al., 2006, Mensi et al., 2012, Cook et al., 2007, Rössert et al., 2017) and is in line with the finding that linear processing of input spike counts accounts for ∼80% of the variance in the mean neuronal firing rate (Poirazi et al., 2003b). However, these previous results were based on simple input patterns, such as constant current injection (Jolivet et al., 2006, Mensi et al., 2012, Cook et al., 2007) or constant input firing rates (Poirazi et al., 2003b), or model inputs that were not calibrated on intracellular recordings (and, e.g., had almost no correlations; Rössert et al., 2017), both of which may lead to an underestimation of the effect of nonlinearities (Figures S1 and S7). In contrast, we estimated the contribution of dendritic nonlinearities under *in vivo*-like input conditions, after carefully calibrating our inputs to intracellular recordings (Figure 2).

While it is possible that spatiotemporal synaptic patterns that we have not tested might lead to different integration regimes, for the *in vivo*-like input patterns analyzed in this study, the main contribution of dendritic mechanisms was to change the gain of linear input integration. We believe that this is because the dominant nonlinearity in our biophysical models was current flowing through NMDARs, which can exhibit different input amplification regimes (Schiller and Schiller, 2001) and produce graded amplification of inputs (Branco and Häusser, 2011). This property makes NMDARs particularly suited to control the gain of synaptic integration and therefore be captured by linear integration processes. In agreement, the effective synaptic kernels we recovered in the hLN clearly reflected the recruitment of NMDARs in the active biophysical model. Beyond these linear effects, the single, global nonlinearity that we found in the best-fitting hLN model also reflects a dendritic processing step, such as the global dendritic nonlinearity recently observed experimentally in L5 and CA1 pyramidal neurons (Xu et al., 2012, Bittner et al., 2015, Takahashi et al., 2016), which could thus be a major mode of dendritic computation *in vivo*.

Importantly, our results demonstrate that simplified single-neuron models can accurately capture input-output transformations of complex nonlinear neurons and therefore be used for studying computation in neuronal networks that are biologically plausible (Dayan and Abbott, 2001).

### Input Multiplexing

In addition to estimating the contribution of nonlinear dendritic processing to the somatic membrane potential, our approach also revealed a novel way of conceptualizing synaptic integration: multiplexing inputs into parallel processing channels with different linear and nonlinear integration properties. Similar multiplexing architectures have been previously applied to model input processing by separate subnetworks during phase-invariant neuronal responses of complex cells in the visual system (Adelson and Bergen, 1985, Rust et al., 2005, Vintch et al., 2015). This form of nonlinear input integration, which significantly increased the accuracy of our model predictions, represents a major transition from conventional models of dendritic processing.

Importantly, multiplexing also provided an interesting biological insight: that the separate fast and slow processing channels enable neurons to dynamically adjust the properties of integration depending on the input statistics. At low input rates, the fast channel has a high gain (Figure 6E), and therefore its output dominates the neuronal response, while the contribution of the slow channel is relatively small. Conversely, the greater curvature of the slow channel (Figure 6H) implies that at higher input rates its gain increases and thus dominates the response, whereas the fast channel becomes saturated (Figure 6E). This arrangement significantly improved the ability of the hLN model to capture the dynamics of NMDAR-dependent integration. The finding that most inhibition was processed via the slow, supralinear channel reflected the increased depolarization and consequently increased driving force for GABAergic currents during the engagement of the slow excitatory channel. These results demonstrate the ability of multiplexing to capture important biophysical effects and suggest that this approach will be useful for abstracting the compound effects of multiple conductances with different dynamic properties, without having to model them explicitly.

### Aspects of Dendritic Processing Not Captured by hLN Models

While in cortical pyramidal cells NMDAR activation has been shown to be the primary influence on neuronal responses *in vivo* (Lavzin et al., 2012, Smith et al., 2013, Palmer et al., 2014, Schmidt-Hieber et al., 2017), which our hLN model captured accurately, future developments of the hLN approach could improve its ability to capture events such as the initiation and propagation of dendritic Na^+^ spikes. In particular, the strong negative correlation between the frequency of dendritic Na^+^ spikes and the performance of the hLN model (Figures S5G and S7J) indicates that most of the unexplained variance arises from dendritically evoked Na^+^ spikes appearing as spikelets of variable amplitudes in the soma (Losonczy and Magee, 2006, Smith et al., 2013, Schmidt-Hieber et al., 2017). This behavior may be better captured, for example, by extending the instantaneous nonlinearities employed here with simplified dynamical models of spike generation and propagation along the network of the hierarchical subunits, as proposed recently for modeling neurons with dendritic calcium spikes (Naud et al., 2014). Interestingly, this observation also suggests that Na^+^ and Ca^2+^ spikes may involve hierarchical processing within the dendritic tree that is fundamentally different from processing NMDA-related nonlinearities. Future extensions of our work should allow simplified descriptions of strongly nonlinear and dynamical forms of dendritic processing proposed by previous theories, including nexus spikes and bursting in L5 pyramidal neurons (Larkum et al., 2001), coincidence detection (Xu et al., 2012, Kaifosh and Losonczy, 2016, Guerguiev et al., 2017), and frequency-modulated ongoing dendritic oscillations (Lengyel et al., 2003, Remme et al., 2010).

We focused on the contributions of dendritic processing to the subthreshold somatic membrane potential, which determines the instantaneous firing rate of the cell (Carandini and Ferster, 2000), as well as its spike count—the measure of neuronal activity considered most relevant for circuit-level computations (London et al., 2010). Extending the hLN with a Poisson spike-generation mechanism allowed prediction of spike timings, albeit with limited precision (Figure S4), which could be improved using more accurate models of spike generation. There also remains the possibility that the 5%–10% unexplained variance in the subthreshold *V*_m_ may correspond to membrane potential dynamics that are important for spike timings and network function. This question can be addressed in future work using hLN models in network simulations that aim to reproduce experimentally recorded network dynamics.

Finally, the function of dendritic voltage-gated ion channels may go far beyond influencing the overall neuronal input-output transformation investigated here. Dendritic spikes, whether global or local, have a major role in controlling synaptic plasticity (Golding et al., 2002, Remy and Spruston, 2007, Kim et al., 2015, Bittner et al., 2015). Active dendritic mechanisms are also involved in a number of highly compartmentalized processes within individual dendrites, such as regulating local Ca^2+^ concentration (Weber et al., 2016) or excitability of dendritic branches (Makara et al., 2009), potentially controlling Hebbian (Cichon and Gan, 2015) and homeostatic synaptic plasticity mechanisms (Branco et al., 2008) on a fine spatial scale. Capturing these local effects should also be possible with a systematic approach similar to ours, which remains agnostic as to the detailed biophysical mechanisms and instead focuses on the effective statistical relationship between a set of physiologically relevant variables (e.g., local Ca^2+^ concentration and measures of plasticity). Such descriptive models could be estimated by statistically principled methods, albeit potentially using a different class of architectures than that of our hLN model.

## STAR★Methods

### Key Resources Table

**Table:** 

REAGENT or RESOURCE	SOURCE	IDENTIFIER
**Deposited Data**		

Code for simulating the biophysical model, generating the inputs, and simulating and fitting hLN models	GitHub	https://github.com/bbujfalussy/hGLM

**Software and Algorithms**		

Neuron (biophysical model)	Hines and Carnevale, 1997	https://www.neuron.yale.edu/neuron/
Python (biophysical model)	Rossum, 1995	https://www.python.org
R (hLN model and data analysis)	R Development Team, 2007	https://www.r-project.org

**Other**		

*In vivo*, V1 L2/3 dendritic recordings	Smith et al., 2013	https://doi.org/10.1038/nature12600

### Contact for Reagent and Resource Sharing

As Lead Contact, Balázs B. Ujfalussy is responsible for all reagent and resource requests. Please contact Balázs B. Ujfalussy at balazs.ujfalussy@gmail.com with requests and inquiries.

### Method Details

#### Biophysical models

Simulations were performed with the NEURON simulation environment (Hines and Carnevale 1997 version 7.4) embedded in Python 2.7 (Rossum, 1995). For the model reported in the main text, we used a detailed reconstruction of a biocytin-filled layer 2/3 pyramidal neuron (NeuroMorpho.org ID Martin, NMO-00904) as described previously (Smith et al., 2013). Briefly, the passive parameters were *C*_m_ = 1 *μ*F/cm^2^, *R*_m_ = 7,000 Ωcm^2^, *R*_i_ = 100 Ωcm, yielding a somatic input resistance of 70 MΩ.

Active conductances were added to all dendritic compartments and occasionally to the soma (Figure 2, Figure S4C) and included the following: voltage-activated Na^+^ channels (soma 100 mS/cm^2^, dendrite 8 mS/cm^2^ and hotspots 60 mS/cm^2^, Nevian et al., 2007); voltage-activated K^+^ channels (10 mS/cm^2^ soma and 0.3 mS/cm^2^ dendrite); M-type K^+^ channels (soma 0.22 mS/cm^2^ and dendrite 0.1 mS/cm^2^); Ca^2+^-activated K^+^ channels (soma 0.3 mS/cm^2^ and dendrite 0.3 mS/cm^2^); high-voltage activated Ca^2+^ channels (soma 0.05 mS/cm^2^ and dendrite 0.05 mS/cm^2^) and low-voltage activated Ca^2+^ channels (soma 0.3 mS/cm^2^ and dendrite 0.15 mS/cm^2^). To accelerate the simulation of the biophysical model we did not model changes in the intracellular Ca^2+^ concentration and kept it at a constant 0.05 μM value. For the simulations shown in Figure 6B-H all active currents were excluded except NMDA receptors.

AMPA, NMDA and GABA-A synapses were modeled as a bi-exponential function, with time constants of AMPA *τ*_1_ = 0.1 ms, *τ*_2_ = 2 ms; NMDA *τ*_1_ = 3 ms, *τ*_2_ = 40 ms and GABA-A *τ*_1_ = 0.1 ms, *τ*_2_ = 4 ms and with the excitatory (inhibitory) reversal potential set to 0 mV (−80 mV), respectively. The Mg^2+^ block of NMDA synapses was modeled according to Jahr and Stevens (1993). The kinetic NMDA receptor model used in Figure 1 was modeled following Kampa et al. (2004) and included five states (unbound, closed, open, slow, and fast desensitization states). To facilitate the comparison with the results using non-kinetic NMDA receptors we assumed the Mg^2+^ block of NMDA synapses to be instantaneous and modeled it according to Jahr and Stevens (1993).

Each excitatory synapse included an AMPA and a NMDA component which were thus colocalized and always coactivated. The maximal conductance of NMDA synapses was set to *g*_max_ = 0.5 nS. The maximal conductance of AMPA synapses were set to *g*_max_ = 0.25 nS, except in Figure 1 where it was varied between *g*_max_ = 0.25 nS (default, NAR = 2) and *g*_max_ = 0.75 (NAR = 0.67), and in Figure 2H where stronger synapses were used (NMDA *g*_max_ = 2 nS and AMPA *g*_max_ = 1 nS) to account for the larger uncaging laser power; and in Figures 6B-6H, where *g*_max_ = 0.75 (NAR = 0.67). The maximal conductance of GABA-A synapses was set to *g*_max_ = 1 nS.

A total of 629 excitatory and 120 inhibitory synapses were uniformly distributed across the entire dendritic tree using the following procedure: an excitatory and an inhibitory synapse was placed at the somatic end of each dendritic branch and further excitatory and inhibitory synapses were added at every 10 μm and 100 μm, respectively. This way the inter synaptic distance was still substantially smaller than the neuronal space constant and thus adding a higher number of proportionally weaker synapses (which might have otherwise been more realistic) would not have substantially altered the integration properties of the biophysical model. An additional set of *N*_soma_ = 420 inhibitory synapses were also added to the soma to model the effect of strong perisomatic inhibition.

To account for the possible depolarization caused by the presence of the recording electrode in the experiments, a small (0.02 nA) constant current was injected to the dendritic branch we monitored in the biophysical model during the simulations shown in Figure 2.

#### Inputs

We modeled the synaptic input of L2/3 pyramidal neurons during the presentation of 16 differently oriented moving grating stimuli by the combination of the following three factors: (1) orientation-dependent cell assembly dynamics; (2) slow fluctuations of firing rates; (3) Poisson spike generation. This input structure was chosen to provide a rich stimulus set that engaged dendritic nonlinearities and matched *in vivo* observed dendritic membrane potential dynamics.

Due to the *in vivo*-like complexity of the input statistics in our model, we used a heuristic procedure to identify initial input parameters that matched experimental data, followed by systematic variation of key parameters (number of excitatory clusters, firing rates, and synchronization of inputs) over a broad range. For each parameter setting, we quantified the quality of the match to experimental data using three summary statistics of dendritic membrane potential fluctuations: overall probability of plateaus, decay time constant of autocorrelations, and distribution of membrane potential values, and chose the best matching parameter set.

To model presynaptic cell-assembly dynamics the excitatory inputs were divided into 13 orientation-tuned functional ensembles, such that inputs within an ensemble were correlated with each other while inputs from different ensembles were independent (Figure 4A). Synapses belonging to a given ensemble were localized on a particular subtree of the entire dendritic tree (Figure 2I) facilitating the generation of dendritic spikes (Polsky et al., 2004) and implementing synaptic clustering (Scholl et al., 2017). Inputs within an ensemble switched randomly and synchronously from a background firing rate (5 Hz) to an elevated activity (20 Hz), where the rate of switching on changed between Ω_on_ = 0.5 Hz and Ω_on_ = 14 Hz as a sine function of stimulus-orientation. The preferred orientation of each dendritic branch (the stimulus orientation corresponding to the maximal on-rate) was randomly chosen from a normal distribution with parameters *μ* = 0° and σ = 33° and then rounded to the nearest multiple of 22.5° (to match the 16 equally spaced stimulus orientations). The duration of the active states had a (truncated) exponential distribution governed by a constant switching off rate, Ω_off_ = 20 Hz, independent of stimulus orientation, with the maximum duration of active states set to 150 ms.

To generate more smoothly varying inputs and to achieve the trial-to-trial variability characteristic of experimental data, we also added a slowly decaying fluctuation component to the excitatory firing rate independently for each ensemble (but shared between inputs belonging to the same ensemble). Specifically, the actual firing rates followed an Ornstein-Uhlenbeck process, decaying toward the state-dependent equilibrium rates (set by the switching process) with a time constant *τ* = 500 ms and having a standard deviation of 2.5 Hz and 10 Hz in the background and in the elevated state, respectively (Ujfalussy et al., 2015). Finally, spikes in the input were generated by an inhomogeneous Poisson process with the rates defined above.

Inhibitory inputs did not show orientation tuning, and were all weakly, but positively correlated with the excitatory inputs (Haider et al., 2013). Their firing rate was proportional to the instantaneous mean of the excitatory firing rates and changed between 20 Hz (when all excitatory ensembles were in the background state) and 30 Hz (all excitatory ensembles being in the elevated state).

Figures 2K-2M shows data averaged over 18 s of activity for each of the 16 different orientations. To train the hLN models, we generated 10 different repetitions (with random state transitions, slow fluctuations and spikes) of 48 s long stimulus blocks consisting of 3 s long sections of each of the 16 orientations.

To demonstrate the robustness of our results, we varied either the input firing rates (Figure S6) or the input correlations (Figure S7). In these figures we only modeled presynaptic assembly dynamics, varying the background and the elevated firing rates of the presynaptic excitatory and inhibitory cells (see table below), but not the orientation selectivity of the inputs or the slow fluctuations in the presynaptic firing rates. The switching on and off rates were Ω_on_ = 1 Hz and Ω_off_ = 10 Hz, respectively.

**Table:** Firing Rates of Presynaptic Inputs in Figure S6

label	background (Hz)		elevated (Hz)		$N_{\text{soma}}$
	excitatory	inhibitory	excitatory	inhibitory	
5	1	5	5	7	420
10	3	10	10	15	420
20	5	20	20	30	420
25	6	55	25	80	100
40	8	80	40	100	100
55	10	150	55	300	100

To analyze the mechanisms underlying input multiplexing, we simulated a simpler scenario where only 160 excitatory and 32 inhibitory inputs distributed on 4 different dendritic branches were stimulated (excitatory, background: 4 Hz, elevated: 24 Hz; inhibitory, background: 14 Hz, elevated: 60 Hz).

#### Hierarchical Linear-Nonlinear (hLN) model

To study the nonlinearity of the input-output transformation of neurons we developed the hierarchical linear-nonlinear (hLN) model which was composed of a cascade of linear and nonlinear processing units (Figure 3). Here we describe the details of the model as well as the procedure we used to fit the model to data.

The collection of input spike trains is represented by a vector s(t), such that $s_{i}\left(t\right)=\sum_{k}\delta \left(t-t_{ik}\right)$, where $t_{ik}$ is the time of the *k*th spike of presynaptic input *i* (*i* = 1…*N*) and δ(⋅) is the Dirac delta function. Each of the M dendritic subunits receives input from $N_{j}$ $\left(\sum_{j}N_{j}=N\right)$ input spike trains through synapses characterized by their time constants, $\tau_{ji}$, propagation delays, ${\text{Δ}}_{ji}$, and synaptic weights, $w_{ji}$, where index *j* and *i* refer to the dendritic subunit and the input, respectively. The total synaptic input, $x_{j}\left(t\right)$ to dendritic subunit *j* is:

Equation 1 $$x_{j}\left(t\right)=\sum_{i\in c_{\text{syn}}\left(j\right)}w_{ji}\int_{0}^{\infty }s_{i}\left(t-t^{\prime }\right)\;\kappa \left(t^{\prime }-{\text{Δ}}_{ji};\tau_{ji}\right)\;\text{d}t^{\prime }$$

Equation 2 $$\;=\sum_{i\in c_{\text{syn}}\left(j\right)}w_{ji}\;\varphi_{ji}$$

where $\varphi_{ji}$ is the total input at a given synapse, $\kappa \left(t;\;\tau_{ji},\;{\text{Δ}}_{ji}\right)$ is the synaptic kernel and $c_{\text{syn}}\left(j\right)$ denotes the set of indices of the synapses connected to subunit *j*. We used the standard alpha function for synaptic kernels:

Equation 3 $$\kappa \left(t;\tau \right)=H\left(t\right)\frac{t}{\tau }e^{-t/\tau }$$

where H(⋅) is the Heaviside step function. We used a combination of two different α-kernels per excitatory synapse for the L2/3 neuron and for both inhibitory and excitatory synapses in the granule cell model. The two kernels were necessary as we found that the functional form of a single alpha synapse was too restrictive to capture linear integration properties of the cells with a mixture of fast and slow synaptic receptors. The two kernels belonged to the same subunit (i.e., sharing the same nonlinearity) and captured linear integration at different timescales. Note that this is different from the multiplexing motif described below, which requires different nonlinearities within the same subunit. The amplitudes of the kernels were independent parameters (*w*^fast^ and *w*^slow^) but we found that their time constants could be coupled through a simple, linear relationship $\tau^{\text{slow}}=10.4+2.8\;\tau^{\text{fast}}\;\left[\text{ms}\right]$ without changing the quality of the fits but decreasing the number of parameters.

When studying input multiplexing (Figure 6), each subunit was allowed to have two different nonlinearities, each of them associated with one (Figures 6B-6H) or two (Figure 6A) α-kernels for each presynaptic spike train. In Figures 6B-6H we allowed only one kernel per nonlinearity in order to highlight the differences between the processing channels.

The total input to a given subunit is the sum of the synaptic inputs and the inputs arriving from other connected subunits:

Equation 4 $$y_{j}\left(t\right)=x_{j}\left(t\right)+\sum_{k\in c_{\text{den}}\left(j\right)}c_{k}\;r_{k}\left(t\right)$$

where where $c_{\text{den}}\left(j\right)$ denotes the set of indices of the subunits connected to subunit j, $c_{k}$ is the strength of coupling of subunit k to its parent and $r_{k}\left(t\right)$ is the activation of subunit k, which is a (logistic) sigmoid function of its total input:

Equation 5 $$r_{j}\left(y;\theta \right)=\frac{1}{1+e^{-\left(y-\theta \right)}}$$

We chose a sigmoid nonlinearity for several reasons. First, the sigmoid has been proposed elsewhere as an appropriate dendritic nonlinearity (Poirazi et al., 2003a, Polsky et al., 2004). Second, under different parameter settings and input statistics, the sigmoid is sufficiently flexible to capture purely linear, sublinear, and supralinear behavior, as well as combinations thereof. The single free parameter of the sigmoid is its threshold, $\theta_{j}$, as its effective slope is set by parameters $w_{ji}$ and $c_{k}$, while its output scale is defined by $c_{j}$. In some simulations, the sigmoid nonlinearity was omitted from the output compartment (e.g., Figure 4C, left) leading to linear integration.

In summary, the response of the hLN model to synaptic inputs is given by

Equation 6 $$\tilde{v}\left(t\right)=c_{1}\;r_{1}\underset{y_{1}\left(t\right)}{\underset{︸}{(\sum_{k\in c_{\text{den}}\left(1\right)}c_{k}\;r_{k}\left(t;\theta_{k}\right)+\underset{x_{1}\left(t\right)}{\underset{︸}{\sum_{i\in c_{\text{syn}}\left(1\right)}w_{1i}\;\varphi_{1i}\left(\tau_{1i},{\text{Δ}}_{1i}\right)}}}};\theta_{1})+v_{0}$$

where the response $\tilde{v}\left(t\right)$ is the output of the hLN model, analogous to the somatic membrane potential, $v_{0}$ is a constant offset, and subunit *k* = 1 refers to the root of the hierarchy. The total number of parameters in a single kernel model with a nonlinear output subunit is thus

Equation 7 $$N_{\text{p}}=1+3N_{\text{syn}}+2M$$

where $N_{\text{syn}}$ is the number of independently fitted synapses and *M* is the number of subunits. Importantly, both the output of the hLN model and its derivative wrt. the parameters can be evaluated in a single sweep starting from the leaves (terminal subunits) and ending at the root subunit. For practical purposes, to avoid overfitting, we tied some of the parameters together by pooling inputs that belonged to the same subunit (see below) so the actual number of fitted synapses *N*_syn_ was lower than the total number of inputs *N*.

In the simulations shown in Figure S4 the model was extended to incorporate somatic spiking (Mensi et al., 2012) which leads to a hierarchical Generalized Linear Model (hGLM). Specifically, the firing rate of the model was an exponential function of the subthreshold response:

Equation 8 $$\lambda \left(t\right)=\lambda_{0}\;\text{exp}\left(\beta \;\tilde{v}\left(t\right)\right)$$

where $\lambda_{0}$ is the baseline firing rate and β describes the sharpness of the firing threshold. Stochastic spikes were generated by an inhomogeneous Poisson process with firing rate λ(t). Somatic spiking triggered adaptation currents which we modeled with an additive term in the membrane potential of the output subunit, such that the total effect of the postsynaptic spikes on the output was a sum of individual adaptation kernels, $\psi_{i}\left(t\right)$, weighted by coefficients $\gamma_{i}$, making the predicted subthrehold response (*cf.* Equation 6)

Equation 9 $$\tilde{v}\left(t\right)=c_{1}\;r_{1}\left(\sum_{k\in c_{\text{den}}\left(1\right)}c_{k}\;r_{k}\left(t;\theta_{k}\right)+\sum_{i\in c_{\text{syn}}\left(1\right)}w_{1i}\;\varphi_{1i}\left(\tau_{1i},{\text{Δ}}_{1i}\right)+\sum_{j=1}^{N_{\psi }}\gamma_{j}\;\psi_{j}\left(t\right);\theta_{1}\right)+v_{0}$$

For the adaptation kernels, we used a set of *N*_*Ψ*_ = 10 basis functions of raised cosine “bumps”, each convolved with the output spike train *s*(*t*):

Equation 10 $$\psi_{i}\left(t\right)=\frac{1}{2}\int s\left(t-\tau \right)\left(\cos\left(a\;\log\left(\tau +c\right)-\phi_{i}\right)+1\right)\;\text{d}\tau$$

for τ such that $a\;\log\left(\tau +c\right)\in \left\{\phi_{i}-\pi ,\phi_{i}+\pi \right\}$ and 0 elsewhere, and a=3.75, c=0.01 and $\phi_{i}$ set uniformly in the interval [3,22] (Pillow et al., 2008).

#### Model fitting procedure

The goal of the model fitting was to match the biophysical model’s somatic membrane potential response, v(t) with the response of the hLN model, $\tilde{v}\left(t\right)$, to the same set of input spike trains.

We assumed in this work that the hLN architecture, defined by the sets $c_{\text{syn}}$ and $c_{\text{den}}$, was given in advance, that is, instead of systematically learning the structure of the model we chose from a couple of preselected candidates based on their ability to predict test data. During fitting, we used gradient descent to minimize the fitting error $\varepsilon =1/T\int_{0}^{T}{\left(v\left(t\right)-\tilde{v}\left(t\right)\right)}^{2}\;\text{d}t$, the mean squared deviation between the subthreshold component of the training signal and the hLN model’s response. The error is a non-convex function of the parameters with multiple local minima. To avoid shallow local minima we first trained simple models and used them to initialize the parameters of more complex models. By using this procedure, simpler models also provided an upper bound on the training error for the more complex models.

Specifically, we first coupled the parameters of all synaptic kernels such that synapses within the excitatory and inhibitory population shared a common amplitude, time constant, and delay and fitted a single subunit-model. We then used the optimized value of these tied parameters as the initial condition for optimizing the parameters of more complex models in which they were not tied any more. This helped us avoid shallow local optima. Next, we initialized models with hierarchical subunit structure and one synapse per subunit by pre-tuning the nonlinearities of the subunits to approximate linear integration with synaptic parameters learned by the simple model. In particular, we rescaled the inputs to the subunits by changing the parameters $w_{ji}$ and *c*_*k*_ such that the distribution of the total input was centered at the central, approximately linear part of the sigmoid nonlinearity with standard deviation 1/ρ, while keeping the somatic response amplitude of individual synapses unchanged (see Equation 11 below). We speculated that if the dendritic nonlinearities are smooth (continuous) functions of the input, than the global optimum of the complex model will be close to the pre-initialised parameters. We repeated this scaling process with various values of ρ (typically in the range [1…8]) and chose the one which resulted in the lowest training error after optimization.

Finally we decoupled the parameters of the synaptic kernels from each other: synapses within each dendritic branch were divided into three groups based on the location of the synapse relative to the stem of the branch (proximal, middle, distal) and only synapses within each group had identical parameters, whereas the time constant and the amplitude of the synaptic kernels was allowed to vary between groups (we used a single synaptic delay for each subunit even in the unconstrained case). Note that the number of synapse-groups was determined by the morphology of the cell and was thus independent of the number of subunits.

In order to prevent overfitting, we used a log-normal prior for the individual response amplitudes and the synaptic time constants in those cases where the number of independently fitted synapses exceeded the number of subunits. Note that in the hLN model the amplitude of the somatic response to the activation of synapse i targeting subunit j is

Equation 11 $$a_{ji}=w_{ji}\;c_{j}\;{r^{\prime }}_{j}\prod_{k\in A\left(j\right)}c_{k}\;{r^{\prime }}_{k}$$

where ${r^{\prime }}_{k}$ is the derivative of the subunit nonlinearity (Equation 5), and, together with the subunit coupling *c*_*k*_, represent the gain of subunit *k*, and A(*j*) denotes the ancestors of subunit *j*, i.e., the subunits located toward the root of the hierarchy. Thus, our prior on *a*_*ji*_ imposes joint constraints on the synaptic weight, the resting slope of the subunit nonlinearity and the subunit couplings. The mean parameter of the prior was set by the parameter values found by the coupled optimization, and the variance parameter was set to match to the variance of the somatic PSPs recorded in response to individual synaptic stimulations in the biophysical model. Synaptic time constants $\tau_{ji}$, delays ${\text{Δ}}_{ji}$ and subunit couplings *c*_*k*_ were log transformed before optimization to ensure positivity.

The parameters of the hLN model were fitted and evaluated on 10 separate segments of 40 s or 48 s long training and test data. Gradients of the error with respect to the model parameters were calculated analytically and optimization was performed using the program R’s built-in “BFGS” method. We quantified the accuracy of the models by the variance explained in the subthreshold signal, which is one minus the fitting error normalized by the variance of the signal:

Equation 12 $$\varepsilon =1-\epsilon /\text{Var}\left[v\left(t\right)\right]$$

The parameters of the spiking response were fitted using the reference spike trains of the biophysical model. The weighting coefficients of the adaptation kernel, *γ*_*i*_, were fitted together with the other subthreshold parameters (Equation 6). To fit the baseline firing rate, *λ*_0_, and the firing rate nonlinearity, β, we maximized the likelihood of the reference spike train given the hLN model’s subthreshold response (Mensi et al., 2012).

### Quantification and Statistical Analysis

#### Statistical details

We ran *n* = 10 independent simulations, each consisting of the randomly generated input and the corresponding output of the biophysical model and the independently fitted set of hLN models, except for Figure S9G where *n* = 16. The goodness of model fit was determined by the variance explained (Equation 12) as described above. When displaying group data, gray lines show individual data points and boxplots show median, quartiles, and range of the data. We used paired t tests to evaluate the significance of the differences between the variances explained by different hLN architectures after confirming the normality of the data using Shapiro-Wilk test.

#### Analysis of the *in vivo* data

Our analysis of *in vivo* dendritic activity is based on a single recording from Smith et al. (2013) that showed clear orientation tuning in its response but no anesthesia-related artifacts (i.e., strong up and down states in the absence of visual stimulation). Our analysis focused on the 3 s long stimulus periods during each of the 6 repetitions of the 16 different stimulus orientations. Plateau probability was calculated as the fraction of time the dendritic membrane potential was above −35 mV. Details of the experimental recording protocol and stimulus presentation can be found in Smith et al. (2013).

The expected dendritic response of the biophysical model (Figures S6D-S6F, S7D-S7F) was calculated by first summing the local dendritic PSPs recorded during individual synaptic stimuli and next linearly scaling the sum to best match the measured response during *in vivo*-like stimulation. The number of dendritic Na^+^ spikes (Figure S6I and Figure S7I) was determined by counting the number of upward zero crossings of the local dendritic membrane potential in the biophysical model.

### Data and Software Availability

The code used for simulating the biophysical model, generating the inputs and simulating and fitting hLN models can be downloaded from github (https://github.com/bbujfalussy/hGLM).

## Supplemental Information

Document S1. Figures S1–S9

Document S2. Article plus Supplemental Information

## References

[bib1] Abrahamsson T., Cathala L., Matsui K., Shigemoto R., Digregorio D.A. (2012). Thin dendrites of cerebellar interneurons confer sublinear synaptic integration and a gradient of short-term plasticity. Neuron.

[bib2] Adelson E.H., Bergen J.R. (1985). Spatiotemporal energy models for the perception of motion. J. Opt. Soc. Am. A.

[bib3] Ahmadian Y., Rubin D.B., Miller K.D. (2013). Analysis of the stabilized supralinear network. Neural Comput..

[bib4] Antolík J., Hofer S.B., Bednar J.A., Mrsic-Flogel T.D. (2016). Model constrained by visual hierarchy improves prediction of neural responses to natural scenes. PLoS Comput. Biol..

[bib5] Behabadi B.F., Polsky A., Jadi M., Schiller J., Mel B.W. (2012). Location-dependent excitatory synaptic interactions in pyramidal neuron dendrites. PLoS Comput. Biol..

[bib6] Bittner K.C., Grienberger C., Vaidya S.P., Milstein A.D., Macklin J.J., Suh J., Tonegawa S., Magee J.C. (2015). Conjunctive input processing drives feature selectivity in hippocampal CA1 neurons. Nat. Neurosci..

[bib7] Branco T., Häusser M. (2011). Synaptic integration gradients in single cortical pyramidal cell dendrites. Neuron.

[bib8] Branco T., Staras K., Darcy K.J., Goda Y. (2008). Local dendritic activity sets release probability at hippocampal synapses. Neuron.

[bib9] Branco T., Clark B.A., Häusser M. (2010). Dendritic discrimination of temporal input sequences in cortical neurons. Science.

[bib10] Carandini M., Ferster D. (2000). Membrane potential and firing rate in cat primary visual cortex. J. Neurosci..

[bib11] Cash S., Yuste R. (1998). Input summation by cultured pyramidal neurons is linear and position-independent. J. Neurosci..

[bib12] Cash S., Yuste R. (1999). Linear summation of excitatory inputs by CA1 pyramidal neurons. Neuron.

[bib13] Cichon J., Gan W.-B. (2015). Branch-specific dendritic ca(2+) spikes cause persistent synaptic plasticity. Nature.

[bib14] Cook E.P., Johnston D. (1997). Active dendrites reduce location-dependent variability of synaptic input trains. J. Neurophysiol..

[bib15] Cook E.P., Guest J.A., Liang Y., Masse N.Y., Colbert C.M. (2007). Dendrite-to-soma input/output function of continuous time-varying signals in hippocampal CA1 pyramidal neurons. J. Neurophysiol..

[bib16] Dayan P., Abbott L.F. (2001). Theoretical Neuroscience.

[bib17] Destexhe A., Rudolph M., Paré D. (2003). The high-conductance state of neocortical neurons in vivo. Nat. Rev. Neurosci..

[bib18] Druckmann S., Banitt Y., Gidon A., Schürmann F., Markram H., Segev I. (2007). A novel multiple objective optimization framework for constraining conductance-based neuron models by experimental data. Front. Neurosci..

[bib19] Druckmann S., Berger T.K., Schürmann F., Hill S., Markram H., Segev I. (2011). Effective stimuli for constructing reliable neuron models. PLoS Comput. Biol..

[bib20] Duguid I., Branco T., London M., Chadderton P., Häusser M. (2012). Tonic inhibition enhances fidelity of sensory information transmission in the cerebellar cortex. J. Neurosci..

[bib21] Farinella M., Ruedt D.T., Gleeson P., Lanore F., Silver R.A. (2014). Glutamate-bound NMDARs arising from in vivo-like network activity extend spatio-temporal integration in a L5 cortical pyramidal cell model. PLoS Comput. Biol..

[bib22] Freeman J., Field G.D., Li P.H., Greschner M., Gunning D.E., Mathieson K., Sher A., Litke A.M., Paninski L., Simoncelli E.P., Chichilnisky E.J. (2015). Mapping nonlinear receptive field structure in primate retina at single cone resolution. eLife.

[bib23] Friedrich P., Vella M., Gulyás A.I., Freund T.F., Káli S. (2014). A flexible, interactive software tool for fitting the parameters of neuronal models. Front. Neuroinform..

[bib24] Gerstner W., Naud R. (2009). Neuroscience. How good are neuron models?. Science.

[bib25] Golding N.L., Spruston N. (1998). Dendritic sodium spikes are variable triggers of axonal action potentials in hippocampal CA1 pyramidal neurons. Neuron.

[bib26] Golding N.L., Staff N.P., Spruston N. (2002). Dendritic spikes as a mechanism for cooperative long-term potentiation. Nature.

[bib27] Grienberger C., Konnerth A. (2012). Imaging calcium in neurons. Neuron.

[bib28] Grienberger C., Milstein A.D., Bittner K.C., Romani S., Magee J.C. (2017). Inhibitory suppression of heterogeneously tuned excitation enhances spatial coding in CA1 place cells. Nat. Neurosci..

[bib29] Guerguiev J., Lillicrap T.P., Richards B.A. (2017). Towards deep learning with segregated dendrites. eLife.

[bib30] Haider B., Häusser M., Carandini M. (2013). Inhibition dominates sensory responses in the awake cortex. Nature.

[bib31] Häusser M. (2001). Synaptic function: dendritic democracy. Curr. Biol..

[bib32] Häusser M., Mel B. (2003). Dendrites: bug or feature?. Curr. Opin. Neurobiol..

[bib33] Häusser M., Spruston N., Stuart G.J. (2000). Diversity and dynamics of dendritic signaling. Science.

[bib34] Hennequin G., Ahmadian Y., Rubin D.B., Lengyel M., Miller K.D. (2018). The dynamical regime of sensory cortex: Stable dynamics around a single stimulus-tuned attractor account for patterns of noise variability. Neuron.

[bib35] Herz A.V., Gollisch T., Machens C.K., Jaeger D. (2006). Modeling single-neuron dynamics and computations: a balance of detail and abstraction. Science.

[bib36] Hines M.L., Carnevale N.T. (1997). The NEURON simulation environment. Neural Comput..

[bib37] Hoffman D.A., Magee J.C., Colbert C.M., Johnston D. (1997). K+ channel regulation of signal propagation in dendrites of hippocampal pyramidal neurons. Nature.

[bib38] Hu H., Martina M., Jonas P. (2010). Dendritic mechanisms underlying rapid synaptic activation of fast-spiking hippocampal interneurons. Science.

[bib39] Huys Q.J.M., Ahrens M.B., Paninski L. (2006). Efficient estimation of detailed single-neuron models. J. Neurophysiol..

[bib40] Jahr C.E., Stevens C.F. (1993). Calcium permeability of the N-methyl-D-aspartate receptor channel in hippocampal neurons in culture. Proc. Natl. Acad. Sci. USA.

[bib41] Jarsky T., Roxin A., Kath W.L., Spruston N. (2005). Conditional dendritic spike propagation following distal synaptic activation of hippocampal CA1 pyramidal neurons. Nat. Neurosci..

[bib42] Jia H., Rochefort N.L., Chen X., Konnerth A. (2010). Dendritic organization of sensory input to cortical neurons in vivo. Nature.

[bib43] Jolivet R., Rauch A., Lüscher H.R., Gerstner W. (2006). Predicting spike timing of neocortical pyramidal neurons by simple threshold models. J. Comput. Neurosci..

[bib44] Kaifosh P., Losonczy A. (2016). Mnemonic functions for nonlinear dendritic integration in hippocampal pyramidal circuits. Neuron.

[bib45] Kampa B.M., Clements J., Jonas P., Stuart G.J. (2004). Kinetics of Mg2+ unblock of NMDA receptors: implications for spike-timing dependent synaptic plasticity. J. Physiol..

[bib46] Keren N., Bar-Yehuda D., Korngreen A. (2009). Experimentally guided modelling of dendritic excitability in rat neocortical pyramidal neurones. J. Physiol..

[bib47] Kim Y., Hsu C.-L., Cembrowski M.S., Mensh B.D., Spruston N. (2015). Dendritic sodium spikes are required for long-term potentiation at distal synapses on hippocampal pyramidal neurons. eLife.

[bib48] Larkum M.E., Zhu J.J., Sakmann B. (1999). A new cellular mechanism for coupling inputs arriving at different cortical layers. Nature.

[bib49] Larkum M.E., Zhu J.J., Sakmann B. (2001). Dendritic mechanisms underlying the coupling of the dendritic with the axonal action potential initiation zone of adult rat layer 5 pyramidal neurons. J. Physiol..

[bib50] Larkum M.E., Waters J., Sakmann B., Helmchen F. (2007). Dendritic spikes in apical dendrites of neocortical layer 2/3 pyramidal neurons. J. Neurosci..

[bib51] Lavzin M., Rapoport S., Polsky A., Garion L., Schiller J. (2012). Nonlinear dendritic processing determines angular tuning of barrel cortex neurons in vivo. Nature.

[bib52] Lazar A.A., Slutskiy Y.B. (2015). Spiking neural circuits with dendritic stimulus processors : encoding, decoding, and identification in reproducing kernel Hilbert spaces. J. Comput. Neurosci..

[bib53] Lengyel M., Szatmáry Z., Érdi P. (2003). Dynamically detuned oscillations account for the coupled rate and temporal code of place cell firing. Hippocampus.

[bib54] London M., Segev I. (2001). Synaptic scaling in vitro and in vivo. Nat. Neurosci..

[bib55] London M., Roth A., Beeren L., Häusser M., Latham P.E. (2010). Sensitivity to perturbations in vivo implies high noise and suggests rate coding in cortex. Nature.

[bib56] Losonczy A., Magee J.C. (2006). Integrative properties of radial oblique dendrites in hippocampal CA1 pyramidal neurons. Neuron.

[bib57] Magee J.C. (2000). Dendritic integration of excitatory synaptic input. Nat. Rev. Neurosci..

[bib58] Major G., Larkum M.E., Schiller J. (2013). Active properties of neocortical pyramidal neuron dendrites. Annu. Rev. Neurosci..

[bib59] Makara J.K., Magee J.C. (2013). Variable dendritic integration in hippocampal CA3 pyramidal neurons. Neuron.

[bib60] Makara J.K., Losonczy A., Wen Q., Magee J.C. (2009). Experience-dependent compartmentalized dendritic plasticity in rat hippocampal CA1 pyramidal neurons. Nat. Neurosci..

[bib61] Mensi S., Naud R., Pozzorini C., Avermann M., Petersen C.C.H., Gerstner W. (2012). Parameter extraction and classification of three cortical neuron types reveals two distinct adaptation mechanisms. J. Neurophysiol..

[bib62] Naud R., Bathellier B., Gerstner W. (2014). Spike-timing prediction in cortical neurons with active dendrites. Front. Comput. Neurosci..

[bib63] Nevian T., Larkum M.E., Polsky A., Schiller J. (2007). Properties of basal dendrites of layer 5 pyramidal neurons: a direct patch-clamp recording study. Nat. Neurosci..

[bib64] O’Keefe J., Recce M.L. (1993). Phase relationship between hippocampal place units and the EEG theta rhythm. Hippocampus.

[bib65] Palmer L., Murayama M., Larkum M. (2012). Inhibitory regulation of dendritic activity in vivo. Front. Neural Circuits.

[bib66] Palmer L.M., Shai A.S., Reeve J.E., Anderson H.L., Paulsen O., Larkum M.E. (2014). NMDA spikes enhance action potential generation during sensory input. Nat. Neurosci..

[bib67] Pecevski D., Buesing L., Maass W. (2011). Probabilistic inference in general graphical models through sampling in stochastic networks of spiking neurons. PLoS Comput. Biol..

[bib68] Petersen C.C.H., Crochet S. (2013). Synaptic computation and sensory processing in neocortical layer 2/3. Neuron.

[bib69] Pillow J.W., Shlens J., Paninski L., Sher A., Litke A.M., Chichilnisky E.J., Simoncelli E.P. (2008). Spatio-temporal correlations and visual signalling in a complete neuronal population. Nature.

[bib70] Poirazi P., Brannon T., Mel B.W. (2003). Arithmetic of subthreshold synaptic summation in a model CA1 pyramidal cell. Neuron.

[bib71] Poirazi P., Brannon T., Mel B.W. (2003). Pyramidal neuron as two-layer neural network. Neuron.

[bib72] Polack P.-O., Friedman J., Golshani P. (2013). Cellular mechanisms of brain state-dependent gain modulation in visual cortex. Nat. Neurosci..

[bib73] Polsky A., Mel B.W., Schiller J. (2004). Computational subunits in thin dendrites of pyramidal cells. Nat. Neurosci..

[bib74] Poulet J.F., Petersen C.C. (2008). Internal brain state regulates membrane potential synchrony in barrel cortex of behaving mice. Nature.

[bib75] R Development Team (2007). R: A language and environment for statistical computing.

[bib76] Ramirez A., Pnevmatikakis E.A., Merel J., Paninski L., Miller K.D., Bruno R.M. (2014). Spatiotemporal receptive fields of barrel cortex revealed by reverse correlation of synaptic input. Nat. Neurosci..

[bib77] Remme M.W., Lengyel M., Gutkin B.S. (2010). Democracy-independence trade-off in oscillating dendrites and its implications for grid cells. Neuron.

[bib78] Remy S., Spruston N. (2007). Dendritic spikes induce single-burst long-term potentiation. Proc. Natl. Acad. Sci. USA.

[bib79] Rössert, C., Pozzorini, C., Chindemi, G., Davison, A. P., King, C. E. J., Newton, T. H., Nolte, M., Ramaswamy, S., Reimann, M. W., Wybo, W., Gewaltig, M.-O., Gerstner, W., Markram, H., Segev, I., and Muller, E. (2017). Automated point-neuron simplification of data-driven microcircuit models. arXiv, arXiv:1604.00087, https://arxiv.org/abs/1604.00087.

[bib80] Rossum G. (1995). Python reference manual. Tech. rep., CWI.

[bib81] Rubin D.B., Van Hooser S.D., Miller K.D. (2015). The stabilized supralinear network: a unifying circuit motif underlying multi-input integration in sensory cortex. Neuron.

[bib82] Rudolph M., Destexhe A. (2003). A fast-conducting, stochastic integrative mode for neocortical neurons in vivo. J. Neurosci..

[bib83] Rust N.C., Schwartz O., Movshon J.A., Simoncelli E.P. (2005). Spatiotemporal elements of macaque v1 receptive fields. Neuron.

[bib84] Schiller J., Schiller Y. (2001). NMDA receptor-mediated dendritic spikes and coincident signal amplification. Curr. Opin. Neurobiol..

[bib85] Schiller J., Schiller Y., Stuart G., Sakmann B. (1997). Calcium action potentials restricted to distal apical dendrites of rat neocortical pyramidal neurons. J. Physiol..

[bib86] Schiller J., Major G., Koester H.J., Schiller Y. (2000). NMDA spikes in basal dendrites of cortical pyramidal neurons. Nature.

[bib87] Schmidt-Hieber C., Toleikyte G., Aitchison L., Roth A., Clark B.A., Branco T., Häusser M. (2017). Active dendritic integration as a mechanism for robust and precise grid cell firing. Nat. Neurosci..

[bib88] Scholl B., Wilson D.E., Fitzpatrick D. (2017). Local order within global disorder: Synaptic architecture of visual space. Neuron.

[bib89] Schwartz O., Pillow J.W., Rust N.C., Simoncelli E.P. (2006). Spike-triggered neural characterization. J. Vis..

[bib90] Silver R.A. (2010). Neuronal arithmetic. Nat. Rev. Neurosci..

[bib91] Smith S.L., Smith I.T., Branco T., Häusser M. (2013). Dendritic spikes enhance stimulus selectivity in cortical neurons in vivo. Nature.

[bib92] Takahashi N., Kitamura K., Matsuo N., Mayford M., Kano M., Matsuki N., Ikegaya Y. (2012). Locally synchronized synaptic inputs. Science.

[bib93] Takahashi N., Oertner T.G., Hegemann P., Larkum M.E. (2016). Active cortical dendrites modulate perception. Science.

[bib94] Truccolo W., Hochberg L.R., Donoghue J.P. (2010). Collective dynamics in human and monkey sensorimotor cortex: predicting single neuron spikes. Nat. Neurosci..

[bib95] Ujfalussy B.B., Makara J.K., Branco T., Lengyel M. (2015). Dendritic nonlinearities are tuned for efficient spike-based computations in cortical circuits. eLife.

[bib96] Urban N.N., Barrionuevo G. (1998). Active summation of excitatory postsynaptic potentials in hippocampal CA3 pyramidal neurons. Proc. Natl. Acad. Sci. USA.

[bib97] Vervaeke K., Lorincz A., Nusser Z., Silver R.A. (2012). Gap junctions compensate for sublinear dendritic integration in an inhibitory network. Science.

[bib98] Vintch B., Movshon J.A., Simoncelli E.P. (2015). A convolutional subunit model for neuronal responses in macaque v1. J. Neurosci..

[bib99] Weber J.P., Andrásfalvy B.K., Polito M., Magó Á., Ujfalussy B.B., Makara J.K. (2016). Location-dependent synaptic plasticity rules by dendritic spine cooperativity. Nat. Commun..

[bib100] Williams S.R. (2004). Spatial compartmentalization and functional impact of conductance in pyramidal neurons. Nat. Neurosci..

[bib101] Wu M.C.-K., David S.V., Gallant J.L. (2006). Complete functional characterization of sensory neurons by system identification. Annu. Rev. Neurosci..

[bib102] Xu N.L., Harnett M.T., Williams S.R., Huber D., O’Connor D.H., Svoboda K., Magee J.C. (2012). Nonlinear dendritic integration of sensory and motor input during an active sensing task. Nature.

[bib103] Zador A.M. (2000). The basic unit of computation. Nat. Neurosci..

