# Supplementary material for: Global and Multiplexed Dendritic Computations under In Vivo-like Conditions
Source: Neuron. 2018 Nov 7;100(3):579–592.e5. doi: 10.1016/j.neuron.2018.08.032 (PMC6226578; doi:10.1016/j.neuron.2018.08.032)
Supplement: Document S1. Figures S1–S9 [file mmc1.pdf]

Neuron, Volume *100*

Supplemental Information

Global and Multiplexed Dendritic Computations under *In Vivo*-like Conditions

Balázs B. Ujfalussy, Judit K. Makara, Máté Lengyel, and Tiago Branco

Global and multiplexed dendritic computations under *in vivo*-like conditions

Balázs B Ujfalussy[1-3,6,8,*], Judit K Makara[2], Máté Lengyel[3,4,7] and Tiago Branco[1,5,7]

[1] MRC Laboratory of Molecular Biology, Cambridge, UK
[2] Laboratory of Neuronal Signaling, Institute of Experimental Medicine, Budapest, Hungary
[3] Computational and Biological Learning Lab, Department of Engineering, University of Cambridge, UK
[4] Department of Cognitive Science, Central European University, Budapest, Hungary
[5] Sainsbury Wellcome Centre, University College London, UK
[6] MTA Wigner Research Center for Physics, Budapest, Hungary
[7] Senior author
[8] Lead Contact
[*] Correspondence: balazs.ujfalussy@gmail.com

Supplemental Figures

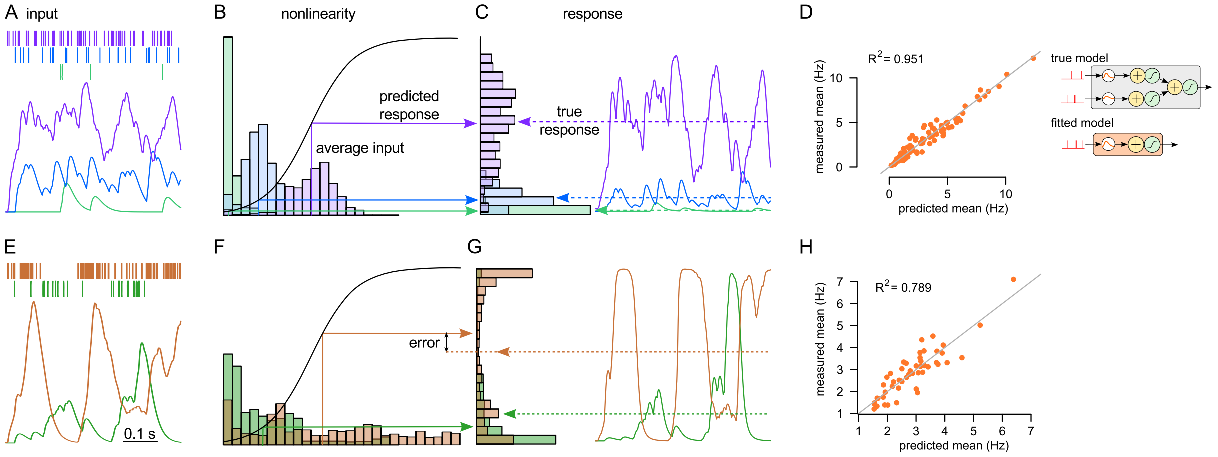

Figure S1. Constant firing rate inputs can mask true dendritic nonlinearities. (Related to Figure 1.)
(A-C) The average response of an LN subunit is accurately predicted by the average input when the inputs have constant firing rates (homogeneous Poisson processes). Input spike trains with different firing rates (A, top, colored rasters) are filtered through a synaptic kernel (A, bottom, filtered traces) and transformed by a sigmoid nonlinearity (B, black curve) to produce the response (C, colored traces). Colored histograms show the distribution of inputs (B) and responses (C). Colored arrows show the response predicted by the average input (B) and the actual average response (C). As each input uses only a modest range of the nonlinearity, the average input accurately predicts the average response.
(D) To illustrate the accuracy of firing rate prediction for constant input firing rates, we stimulated a 2-layer hLN model (inset, top) with a wide range of input firing rates (5-500 Hz) held constant over each 1-s trial, and measured its average output firing rate over the trial. We fitted a one-layer model without local nonlinearities (but with a global nonlinearity, inset, bottom). We found that this simple model, implying linear dendritic processing, predicted the response of the true model, including strong local nonlinearities, with high accuracy ($R^2 = 0.95$).
(E-G) Same as A-C but predicting the response of an LN unit to fluctuating stimuli. When the inputs exhibit strong fluctuations (E), the average input (F) is not predictive of the average output (G), resulting in a substantial prediction error (F-G, arrows).
(H) Same as D but for fluctuating inputs. The one-layer model is less accurate in predicting the firing rate of the same 2-layer hLN model as in D ($R^2 = 0.79$). Fluctuating input was generated by randomly resampling the input rate from the 5-500 Hz interval every 100 ms. In the simulations shown in panels D and H a constant 150 Hz background input was also added to model noisy input processing or unobserved stimuli, but this did not change the results qualitatively.

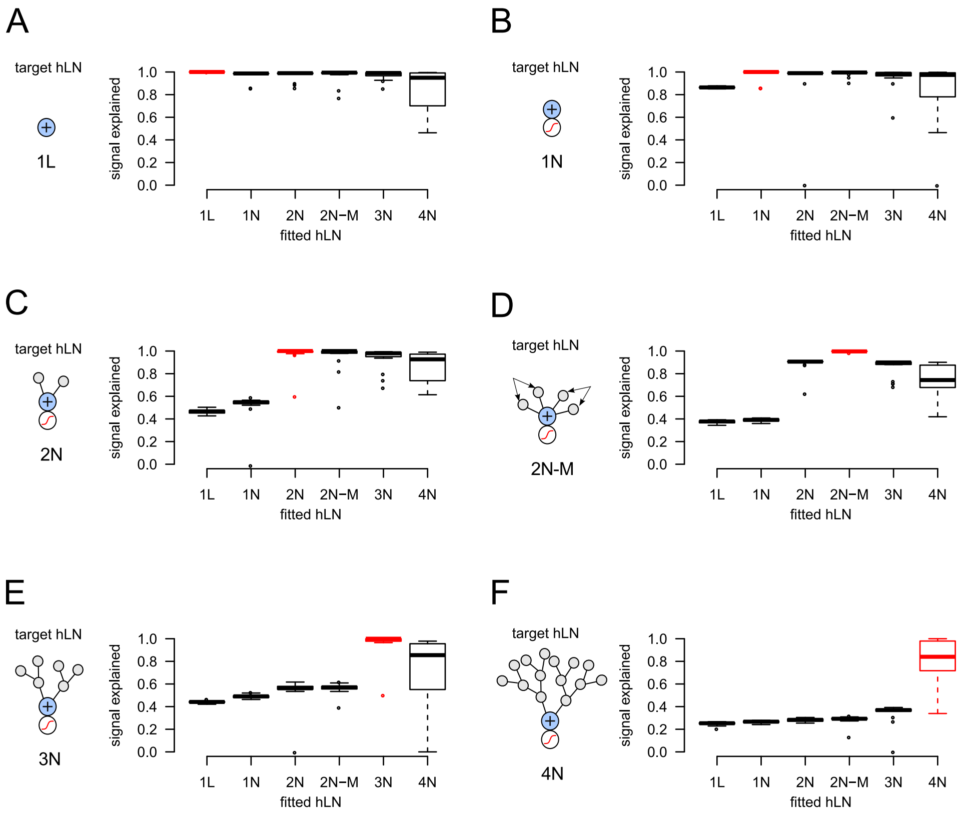

Figure S2. Validation of the model fitting procedure. (Related to Figure 3.) We generated synthetic data by simulating the response of target hLN models with increasingly more complex architectures to random (Poisson) input spike trains. Schematics in panels A-F show the architectures of the target hLN; the name of the model indicates the number of layers in the hierarchy (1-4), the presence or absence of an output nonlinearity (L or N) and multiplexing dendrites, M in panel D. We fitted each synthetic data set (consisting of the inputs and responses of the target hLN models) using hLN models with various architectures (x-axis, the same set of models as that of the targets used to generate data) starting from random initial parameters. The quality of the fits was evaluated by the signal explained ($1-\sqrt{1-\text{variance explained}}$) on a held out test data set. We found that in almost all cases the structure of the original model could be correctly recovered by taking the simplest amongst models achieving maximal performance, indicated by the red color. Boxplot shows median, quartiles and the range of 20 independent simulations, each using a different randomly sampled set of parameters for the target model.

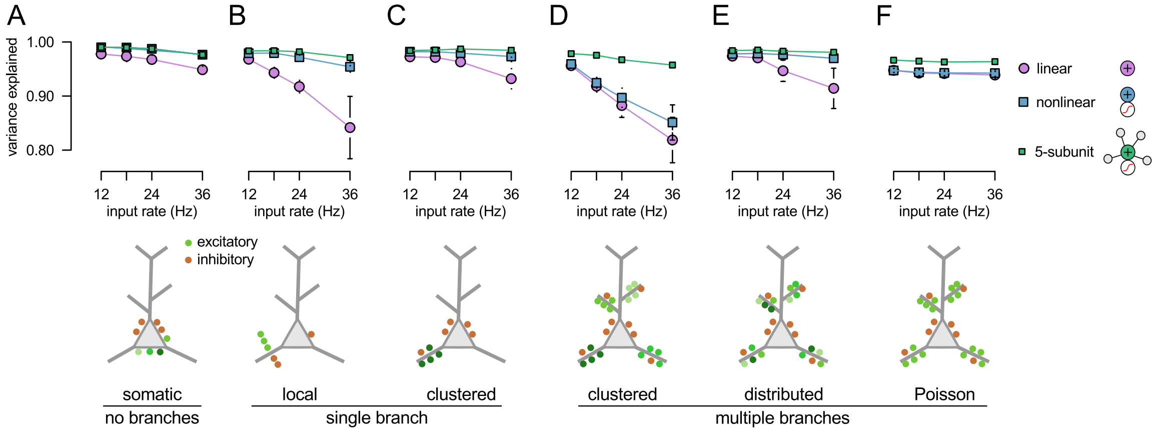

Figure S3. Validation of hLN model assumptions. (Related to Figure 3.) To rigorously validate the ability of the hLN model class to correctly capture the integration of spatially distributed inputs, despite its drastic discretization of the cell's morphology into a small number of independent subunits, we conducted a series of simulation experiments varying the spatio-temporal complexity of the inputs. Specifically, we varied the location of the inputs as follows (see schematics below A-F): (A) all excitatory and inhibitory synapses were placed at the soma; (B) a single presynaptic ensemble targeted the same location within a single dendritic branch; (C) similar to B, but synapses were distributed along the whole length of a single dendritic branch; (D) four presynaptic ensembles targeted 4 dendritic branches in a clustered way: inputs belonging to the same ensemble were distributed along the whole length of a single dendritic branch (as in Figure 6B); (E) similar to D, but inputs were randomly assigned to branches; (F) four branches were targeted, but the inputs were uncorrelated (Poisson spike trains with constant firing rate). Synaptic input patterns were similar to the inputs used in Figure 6B-H, except that in each condition we varied the peak excitatory firing rate between 12 and 36 Hz (x-axis) and we used only a single presynaptic ensemble in panel B and uncorrelated inputs in panel F. The response of a passive biophysical model expressing only NMDA nonlinearities was fitted by three different hLN architectures (insets in A): a "point neuron" with a single linear subunit (linear); a "point neuron" with a single nonlinear subunit (nonlinear); and a neuron with 5 nonlinear subunits (5-subunit). Note, that for panels A-C some subunits of the 5-subunit model did not receive inputs.

We found that the model in which the subunits corresponded to individual dendritic branches always achieved excellent performance ($>$ 95% variance explained). Specifically, the assumption that dendritic branches correspond to discrete integrative compartments (subunits) under these simplified input conditions is justified by a similar fraction of variance being explained when inputs are distributed along the whole dendritic branch (C) versus when they target a single location within the dendritic branch (B) or when dendritic nonlinearities are bypassed by placing all synapses directly on the soma (A). Moreover, the assumption of unidirectional coupling between the subunits is supported by the similar fraction of variance explained by the 5-subunit model when inputs are targeting a single (C) versus multiple dendritic branches (D-F). Error bars (often smaller than the symbols) show standard deviation across 10 independent simulations.

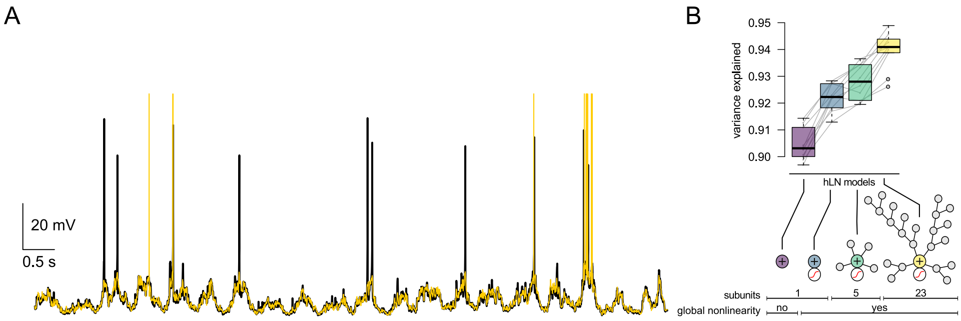

Figure S4. Model with somatic spiking. (Related to Figure 4.)
(A) Somatic membrane potential response of the biophysical model L2/3 neuron to similar input as in Figure 4 with somatic spiking conductances (black) and the sub- and suprathreshold activity predicted by a hierarchical model with 23 subunits (yellow).
(B) Subthreshold variance explained by different hierarchical models when predicting the activity of a biophysical model with somatic spiking. Note that the hGLM model's subthreshold prediction accuracy did not decrease despite the effect of the back-propagating action potentials on dendritic integration in the biophysical model (cf. Figure 4). Boxplots show median, quartiles and the range of 10 independent simulations, open circles indicate outliers.

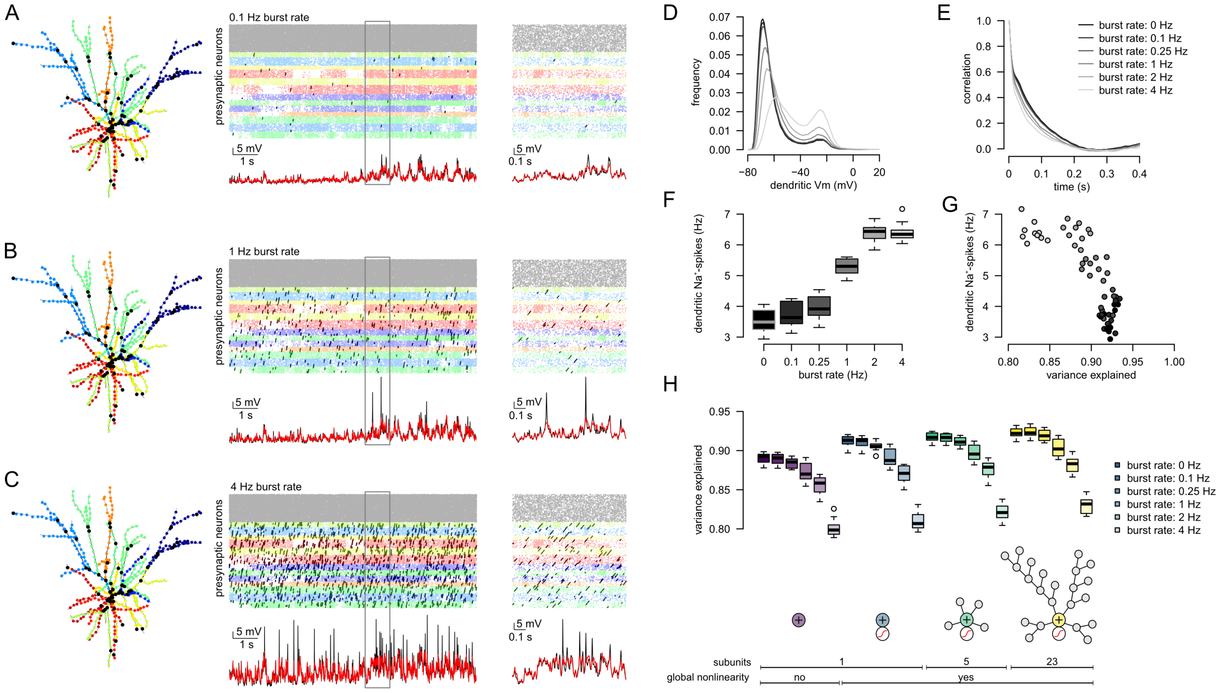

Figure S5. Dependence of dendritic input-output transformation on input synchronization. (Related to Figure 4.)
(A-C) Synaptic clusters (left, color coded) and synaptic inputs (top, color code as in Figure 4A) and somatic output (bottom, black) when synchronous bursts (dark clusters of spikes, 5-30 neighouring synapses activated with 1 ms inter-spike interval) were embedded in the *in vivo*-like synaptic input pattern (lighter colored spikes, same statistics as in Figure 4A). Red line (bottom) shows the response predicted by a hLN model with 23 subunits. Input bursts increased the firing rate with 0.1 (A), 1 (B) or 4 Hz (C) of each input spike train (burst rate). Insets on the right show 1 s of the data on extended time scale.
(D-E) Histogram (D) and auto-correlation (E) of the dendritic membrane potential at different burst rates (shades of grey). In the biologically relevant input range the histogram is slightly bimodal and the autocorrelation has a slow decay (cf. Figure 2F-G, data recorded from a different dendritic branch).
(F) Frequency of dendritic Na^+-spikes in a dendritic branch of the biophysical model as a function of the input burst rate.
(G) Frequency of dendritic Na^+-spikes in a representative dendritic branch of the biophysical model as a function of the variance explained by the 23-subunit hLN model for different values of input burst rate (shades of grey as in D-F).
(H) Variance explained by hLN models with increasing complexity (same as in Figure 4C) as a function of input burst rate.
Boxplots in F and H show median, quartiles and the range of 10 independent simulations, open circles indicate outliers. For reference, other simulations had 0 Hz input burst rate.

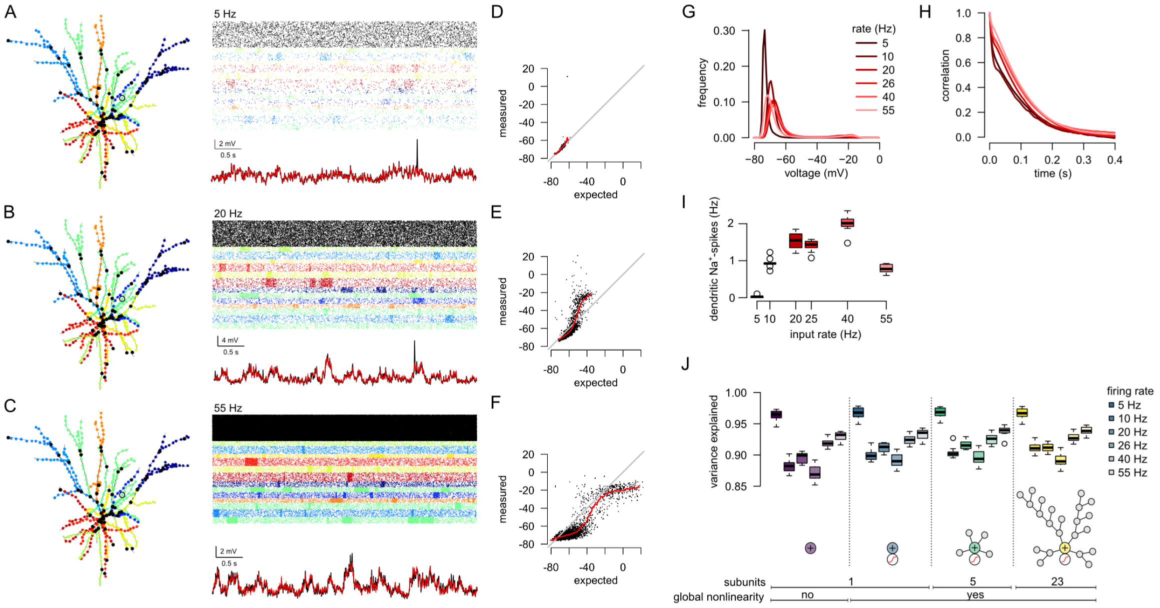

Figure S6. Dependence of dendritic input-output transformation on input firing rate. (Related to Figure 4.)
(A-C) Synaptic clusters (left, color coded) and synaptic inputs (top, color code as in Figure 4A) and somatic output (bottom, black) with excitatory input peak firing rates increasing from 5 to 55 Hz (Table 1). Red line (bottom) shows the response predicted by a hLN model with 23 subunits.
(D-F) Measured dendritic membrane potential as a function of the expected response. Black dots: individual data point, red line: running average trend line.
(G-H) Histogram (G) and auto-correlation (H) of the dendritic membrane potential at different input rates (color). In the biologically relevant input range the histogram is bimodal and the autocorrelation has a slow decay (cf. Figure 2F-G).
(I) Frequency of dendritic spikes in a representative dendritic branch of the biophysical model as a function of the input firing rate.
(J) Variance explained by hLN models with increasing complexity (same as in Figure 4C) as a function of the input firing rate. The performance of linear models (1 subunit) is high even when the dendrites show substantial nonlinearity (e.g., at 55 Hz in panel F).
Boxplots in I and J show median, quartiles and the range of 10 independent simulations, open circles indicate outliers. For reference, simulations in Figures 2, 4, S5 and S7 used ~20 Hz input rates.

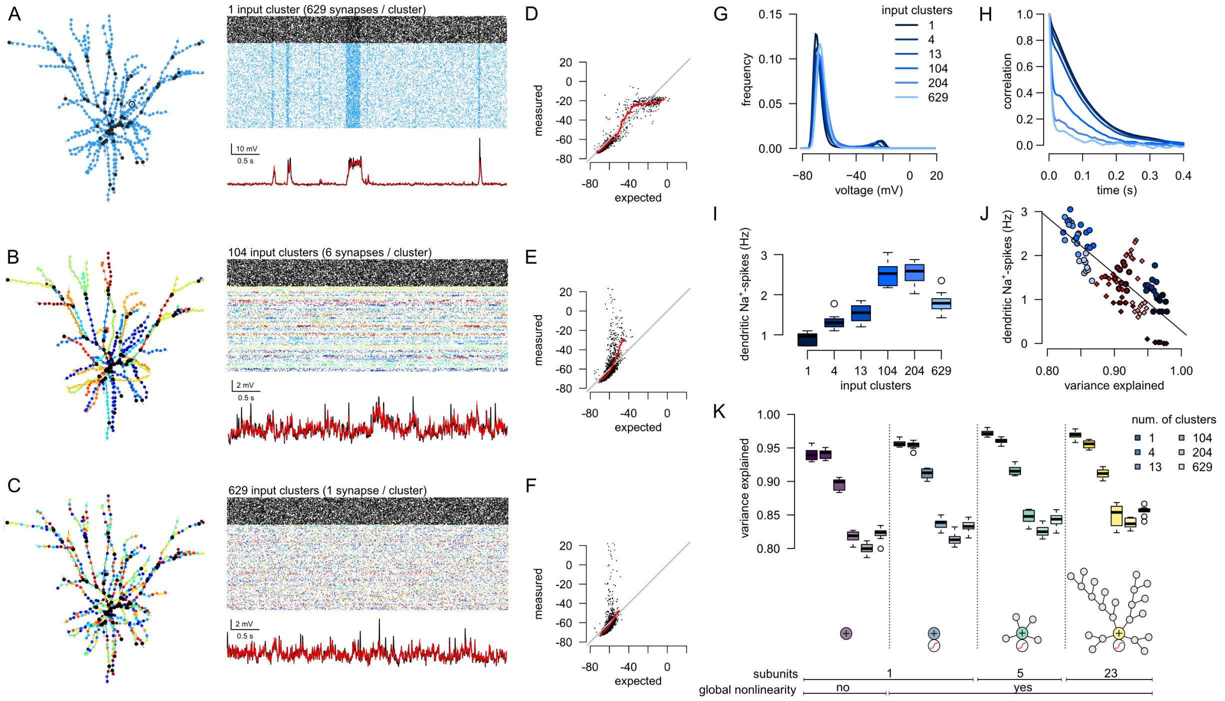

Figure S7. Dependence of dendritic input-output transformation on input clustering. (Related to Figure 4.)
(A-C) Functional synaptic clusters (left, color coded), and inputs (top) together with the somatic output (bottom) with the number of synaptic clusters increasing from 1 (all synapses belong to the same cluster) to 629 (all synapses are independent). Red line (bottom) shows the response predicted by a hLN model with 23 subunits.
(D-F) Measured dendritic membrane potential as a function of the expected response. Black dots: individual data points, red line: running average trend line.
(G-H) Histogram (G) and auto-correlation (H) of the dendritic membrane potential at different levels of synaptic clustering (color). In the biologically relevant input range the histogram is bimodal and the autocorrelation has a slow decay (cf. Figure 2F-G).
(I) Frequency of dendritic Na^{+}-spikes in a representative dendritic branch of the biophysical model as a function of synaptic clustering.
(J) Frequency of dendritic Na^{+}-spikes in a representative dendritic branch of the biophysical model as a function of the variance explained by the 23-subunit hLN model for different values of synaptic clustering (circles, shades of blue, as in panel I) and input firing rate (diamonds, shades of red, as in Figure S6I). The strong negative correlation indicates that most of the unexplained variance arises from dendritically evoked Na^{+}-spikes appearing as spikelets of variable amplitudes in the soma (see also the voltage traces in panels A-C, Fig. S5A-C and S6A-C).
(K) Variance explained by hLN models with increasing complexity (same as in Figure 4C) as a function of synaptic clustering. The performance of linear models (1 subunit) is high even when the dendrites show substantial nonlinearity (panel D).
Boxplots in I and K show median, quartiles and the range of 10 independent simulations, open circles indicate outliers. For reference, simulations in Figures 2, 4, S5 and S6 contained 13 clusters.

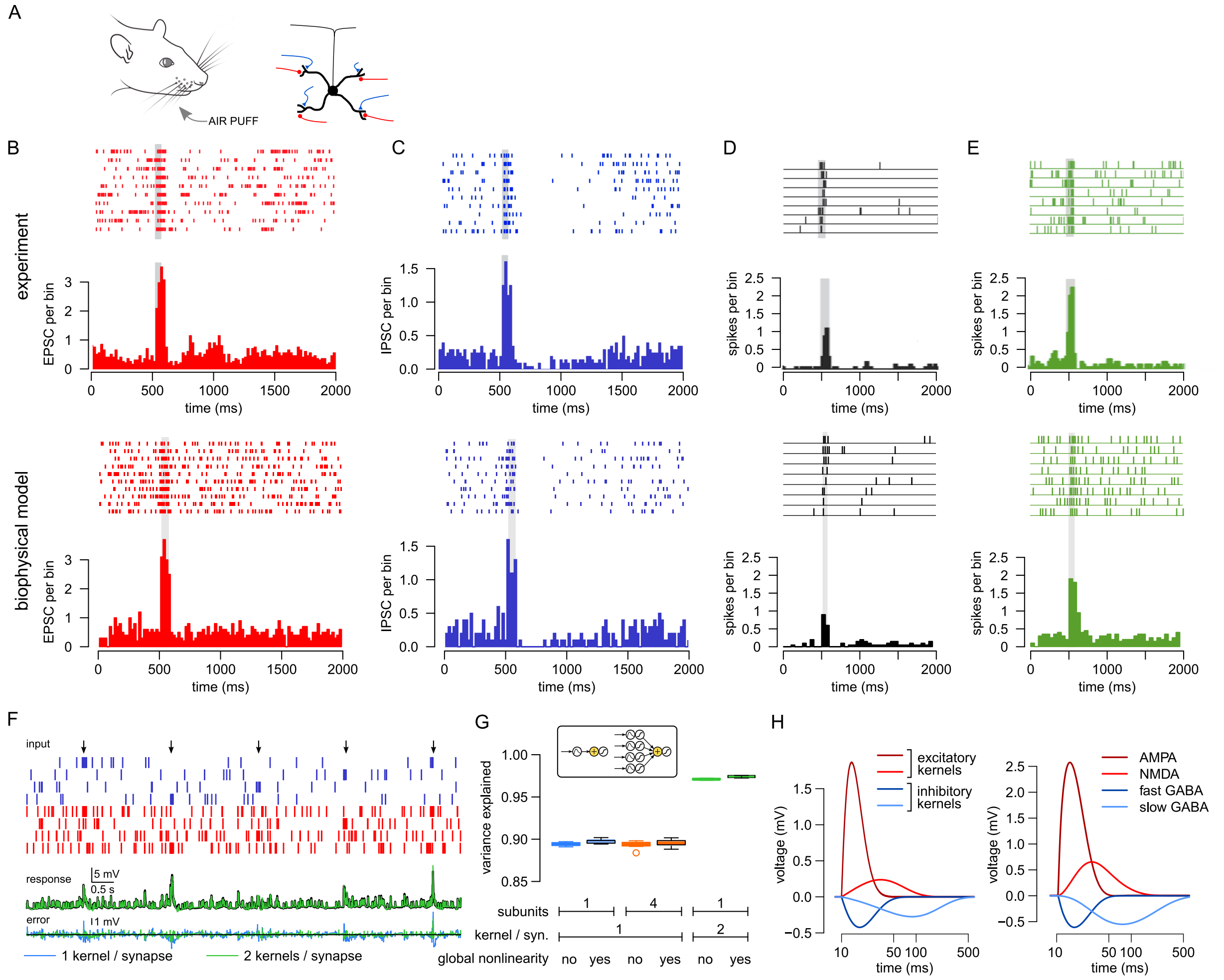

Figure S8. Dendritic integration in cerebellar granule cells *in vivo*. (Related to Figure 4.)
(A) Schematics of the experimental configuration (left) and the granule cell morphology, with excitatory (red) and inhibitory (blue) synaptic contacts made on its four dendritic claws (right).
(B-E) Matching the inputs (B-C) and the output (D-E) of the biophysical model neuron (bottom) to experimental data (top) from Duguid et al. (2015) (B-C) and Duguid et al. (2012) (D-E). B: Raster (ticks) and peri-stimulus time histogram of input EPSPs recorded *in vivo* from cerebellar granule cells (top) together with the synaptic inputs used in the model (bottom). C: IPSPs *in vivo* (top) and in the model (bottom). D: Somatic action potentials under control input conditions recorded *in vivo* (top) and in the model (bottom). E: similar to D but after blocking $GABA_A$ inhibitory inputs with gabazine. Gray shaded area in B-E indicates period of stimulus presentation (facial air puff, see A).
(F) Top: *in vivo*-like synaptic input patterns. Each of the four dendrites receives inputs from a pair of excitatory (red) and inhibitory synapses (blue) with correlated activity. Arrows indicate sensory stimulation. Middle: the subthreshold membrane potential response of the biophysical model (black), and the prediction of the linear hLN model with a mixture of kernels (green). Bottom: error between the response of the biophysical model and the prediction of the linear model with simple (blue) or mixture kernels (green).
(G) Prediction accuracy of hLN models. Insets show model hLN architectures (only excitatory inputs are shown for clarity). Models with simple kernels (blue and orange) have one inhibitory and one excitatory synaptic kernel in each subunit. The multilayered model (orange) has four nonlinear subunits each corresponding to one dendritic branch of the biophysical model. The model with mixture kernels (green) integrates both excitatory and inhibitory inputs with two different synaptic kernels. Each model has two variants: with (box plots with black contour) or without a global output nonlinearity (box plots without black contour). We found that a simple linear model accounted for 90% of variance (F, bottom, blue, and G, blue), with a global nonlinearity improving accuracy only a little (G, blue with black outline). Adding further layers of nonlinear subunits also did not improve accuracy (G, orange). In contrast, introducing a mixture of synaptic kernels for both excitatory and inhibitory inputs improved accuracy to 97% (F, middle and bottom, green, and G, green), with an additional global nonlinearity again contributing little (G, green with black outline). Boxplots show median, quartiles and the range of 10 independent simulations, open circle indicates an outlier.
(H-I) Synaptic kernels recovered from the hLN model with mixture kernels by fitting the *in vivo*-like input-output mapping (left) and individual synaptic responses in the biophysical model (right). Quantitatively, the estimated kernels of the hLN model fitting *in vivo*-like data were somewhat smaller in amplitude and faster than the PSPs in the biophysical model because the increased total membrane conductance during *in vivo*-like conditions rendered membrane potential dynamics more leaky, thus decreasing both the input resistance and time constant of the cell. Note logarithmic time axes.

Model details. The biophysical model of the cerebellar granule cell consisted of a spherical somatic compartment (diameter: $5.8\,\mu$m) connected to four $20\,\mu$m long dendritic branches (diameter: $0.75\,\mu$m), and an axon hillock (d=1.5 μm, L=2.5 μm) continuing as an axon (d=1 μm, L=200 μm). The passive parameters of the model were $C_{\text{m}} = 1\,\mu\text{F/cm}^2$, $R_{\text{m}} = 5000\,\Omega\text{cm}^2$, $R_{\text{i}} = 100\,\Omega\text{cm}$. In the simulations with somatic spiking (Figure S8D-E) voltage-activated Na^+ channels (80 mS/cm^2) and voltage-activated K^+ channels (2 mS/cm^2 soma and hillock, 1.5 mS/cm^2 axon) were also inserted. Each dendritic branch was contacted by one inhibitory (GABA-A and GABA-B) and one excitatory (AMPA and NMDA) synapse, modelled with bi-exponential functions with time constants and maximal conductance of AMPA $\tau_1 = 0.22$ ms, $\tau_2 = 2.5$ ms, $g_{\text{max}} = 0.2$ nS; NMDA $\tau_1 = 3$ ms, $\tau_2 = 40$ ms, $g_{\text{max}} = 0.2$ nS; GABA-A $\tau_1 = 0.39$ ms, $\tau_2 = 6.8$ ms, $g_{\text{max}} = 0.5$ nS and GABA-B $\tau_1 = 30$ ms, $\tau_2 = 150$ ms, $g_{\text{max}} = 0.2$ nS and the excitatory (inhibitory) reversal potential was set to 0 mV (-65 mV), respectively. The application of gabazine was modelled by setting the maximal conductance of GABA-A to 0.

In vivo input during brief air-puff stimulation of the lip area and whiskers (Duguid et al., 2012, 2015) was modelled as an inhomogenous Poisson process with one of the four presynaptic excitatory mossy fibers and inhibitory Golgi cells transiently increasing (mossy fibers: 150 Hz; Golgi cells: 60 Hz, duration: 80 ms) and then decreasing (mossy fibers: 6.25 Hz; Golgi cells: 0.75 Hz, duration: 0.4 s) their firing rates compared to a baseline activation (mossy fibers: 6.25 Hz; Golgi cells: 2.5 Hz). The inter-stimulus interval was 2 s.

References

Duguid, I., Branco, T., Chadderton, P., Arlt, C., Powell, K., & Häusser, M. (2015). Control of cerebellar granule cell output by sensory-evoked golgi cell inhibition. Proc Natl Acad Sci U S A, 112, 13099–104.

Duguid, I., Branco, T., London, M., Chadderton, P., & Häusser, M. (2012). Tonic inhibition enhances fidelity of sensory information transmission in the cerebellar cortex. J Neurosci, 32, 11132–43.

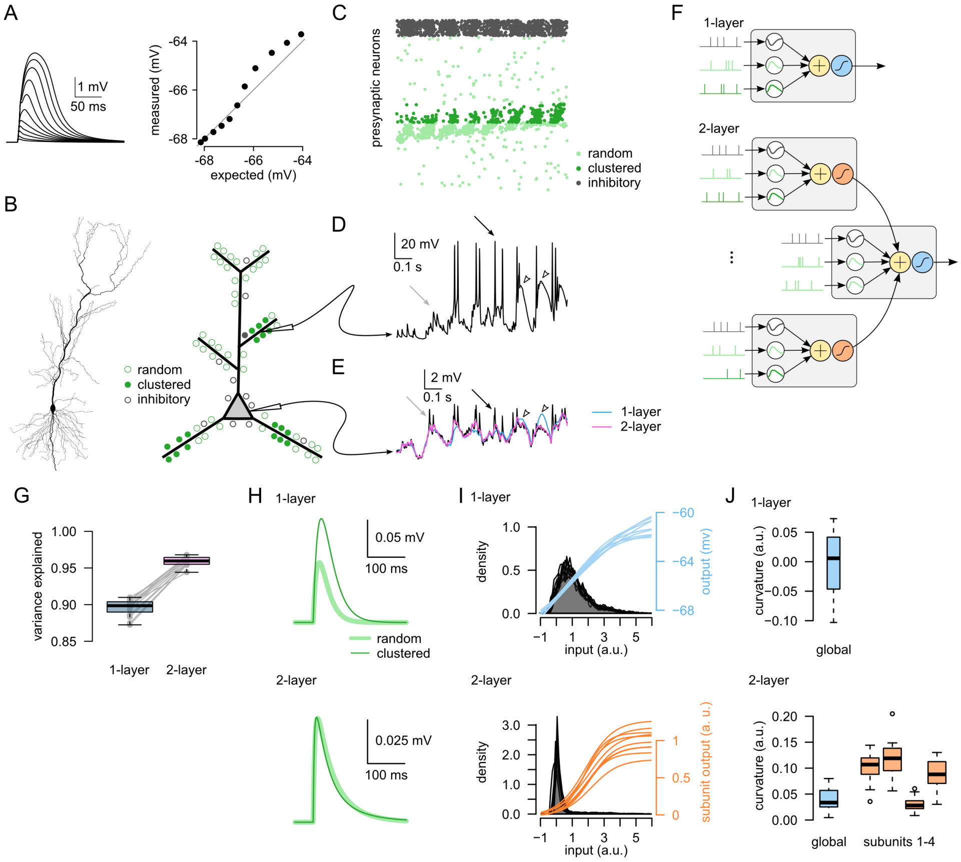

Figure S9. Dendritic integration in CA1 pyramidal neurons *in vivo*. (Related to Figure 4.)
(A) Nonlinear dendritic integration in the biophysical CA1 pyramidal neuron model. Left: somatic responses to an increasing number of synaptic inputs (n= 1, 2, 4, 6, 8, 10, 12, 15, 20, 25, 30) delivered at a single dendritic branch at 0.3 ms intervals. Right: measured response amplitude as a function of the response amplitude expected from linear integration.
(B) The morphology of the biophysical model CA1 neuron used in the simulations (left) and the schematics of the distribution of excitatory (green) and inhibitory (grey) synapses on the model (right). In total, 2000 excitatory synapses were placed on the dendritic tree of which 240 synapses were arranged in 4 functional synaptic clusters on 4 dendritic branches. Additionally, 200 inhibitory synapses were used of which 80 synapses were located at the soma.
(C) *In vivo*-like hippocampal inputs. Spikes from 2000 excitatory cells (green) and 200 inhibitory neurons (grey) were simulated as an inhomogenous Poisson process. The firing rate of the presynaptic population mimicked the activity of cells during the animal's traversal of a 2 m-long linear track, with excitatory neurons exhibiting a single idealised place field, modulated by theta oscillation and undergoing phase precession. Inhibitory neurons were modulated by theta but were spatially untuned. Spikes of neurons with clustered synapses on the dendrites (B) are shown in dark green.
(D) Dendritic membrane potential recorded in one of the branches receiving clustered synaptic inputs. Empty arrowheads highlight dendritic NMDA spikes. Black arrow indicates a local Na^+ spike and grey arrow shows a Na^+ spike propagated from a different dendritic branch.
(E) Measured somatic membrane potential (black) together with the 1-layer (blue) and 2-layer models' prediction (purple, see panel F for model schematics). Note that the somatic response during dendritic NMDA spikes is best captured by the 2-layer model (empty arrowheads). Black and grey arrows highlight dendritic sodium spikes not well captured by the hLN models.
(F) Schematics of the hLN models fitted to data from the CA1 cell. In both models, kernels associated with random (light green) and clustered (dark green) synapses were fitted separately and we used 4 dendritic subunits in the 2-layer model. When fitting the response of the CA1 cell we used double exponential kernels as they resulted in significantly higher performance than the alpha synapses used for the L2/3 cell.
(G) Performance of the 1-layer and 2-layer models evaluated on test data in 16 different runs.
(H-J) Signal processing in the 1-layer (top) and the 2-layer (bottom) hLN models: average synaptic kernels associated with random (H, light green) and clustered inputs (H, dark green), the distribution of linearly integrated inputs (I, gray histograms) relative to the sigmoid of the global (I, blue) or local nonlinearities (I, orange), and the corresponding average curvatures of these nonlinearities (J). The larger effect of clustered inputs on the somatic membrane potential is captured by unequal kernel amplitudes in the 1-layer model (H, top) which uses mostly linear processing (I-J, top). In contrast, in the 2-layer model this is captured by the supralinear transformations associated with the subunits to which clustered inputs arrive (I-J, bottom) without a difference in kernel amplitude (H, bottom).
Boxplots in G and J show median, quartiles and the range of 16 independent simulations, open circles indicate outliers.

Model details. We used a slightly modified version of the CA1 pyramidal cell model of Jarsky et al. (2005) to account for the interactions between dendritic Na^+-spikes and NMDA nonlinearities observed in CA1 pyramidal neurons (Losonczy & Magee, 2006). The model included AMPA and NMDA excitation and slow and fast GABAergic inhibition with parameters AMPA $\tau_1 = 0.1$ ms, $\tau_2 = 1$ ms, $g_{max} = 0.6$ nS; NMDA $\tau_1 = 2$ ms, $\tau_2 = 50$ ms, $g_{max} = 0.8$ nS; $GABA_A$ $\tau_1 = 0.1$ ms, $\tau_2 = 4$ ms, $g_{max} = 0.1$ nS; $GABA_B$ $\tau_1 = 1$ ms, $\tau_2 = 40$ ms, $g_{max} = 0.1$ nS, and voltage gated Na^+, K^+_{DR} and K^+_A channels with the following densities: Na: 30 S/cm^2 axon initial segment, 0.04 S/cm^2 soma, 0.03 S/cm^2 dendrites and increasing from 0.04 S/cm^2 to 0.06 S/cm^2 along the apical trunk between 100 and 500 μm; K_{DR}: 0.04 S/cm^2 axon, 0.04 S/cm^2 soma and apical trunk, 0.02 S/cm^2 dendrites; K_A: 0.004 S/cm^2 axon, 0.02 S/cm^2 soma, 0.02 S/cm^2 in terminal branches and increasing from 0.048 S/cm^2 to 0.29 S/cm^2 along the apical trunk between 100 and 500 μm. The activation curve of the Na^+ channels was shifted by 20 mV in the oblique and tuft branches to account for the similar threshold for Na^+ and NMDA spikes (Losonczy & Magee, 2006). A detailed description of the model will be presented elsewhere (Ujfalussy and Makara, in preparation).

References

Jarsky, T., Roxin, A., Kath, W. L., & Spruston, N. (2005). Conditional dendritic spike propagation following distal synaptic activation of hippocampal CA1 pyramidal neurons. Nat Neurosci, 8, 1667–76.

Losonczy, A. & Magee, J. C. (2006). Integrative properties of radial oblique dendrites in hippocampal CA1 pyramidal neurons. Neuron, 50, 291–307.
